# Supplementary material for: Cooperative genetic networks drive embryonic stem cell transition from naïve to formative pluripotency
Source: EMBO J. 2021 Mar 9;40(8):e105776. doi: 10.15252/embj.2020105776 (PMC8047444; doi:10.15252/embj.2020105776)
Supplement: Supplementary file 2 — Appendix [file EMBJ-40-e105776-s008.pdf]

Appendix

Cooperative genetic networks drive embryonic stem cell transition from naïve to formative pluripotency

Andreas Lackner[#], Robert Sehlke[#], Marius Garmhausen[#], Giuliano Giuseppe Stirparo[#], Michelle Huth[$], Fabian Titz-Teixeira[$], Petra van der Lelij, Julia Ramesmayer, Henry Fabian Thomas, Meryem Ralser, Laura Santini, Elena Galimberti, Mihail Sarov, A. Francis Stewart, Austin Smith, Andreas Beyer*, Martin Leeb*

Appendix Table of Contents

Appendix Figure S1 – Exit from Pluripotency Screen

Appendix Figure S2 – KO validation and rescue experiments

Appendix Figure S3 – RNAseq: Sample clustering dendrograms

Appendix Figure S4 – RNAseq: Batch correction and DEGs

Appendix Figure S5 – *Csnk1a1* KO phenotype

Appendix Figure S6 – 2h resolved time course

Appendix Figure S7 – Pathway and cluster analysis

Appendix Figure S8-S20 – Heatmaps of cluster and GO term genes

A

Gene	times hit in 35 screens	integration sites	FDR
Tcf7l1	34	166	1E-232
Fgfr2	25	78	6.1E-21
Alg13	20	35	9.3E-39
Ctbp2	17	43	5.6E-45
Arfip1	17	29	4.5E-34
Jarid2	16	35	7E-31
Rbpj	16	25	7E-19
Nsd1	14	29	1.1E-26
Arid5b	14	17	1.9E-11
Fbxw7	13	25	1.9E-16
Msi2	13	24	1.2E-12
Dym	13	16	6.6E-07
Etv5	12	26	8E-30
Mapk1	12	19	1.1E-16
Smg7	12	19	3.5E-15

D

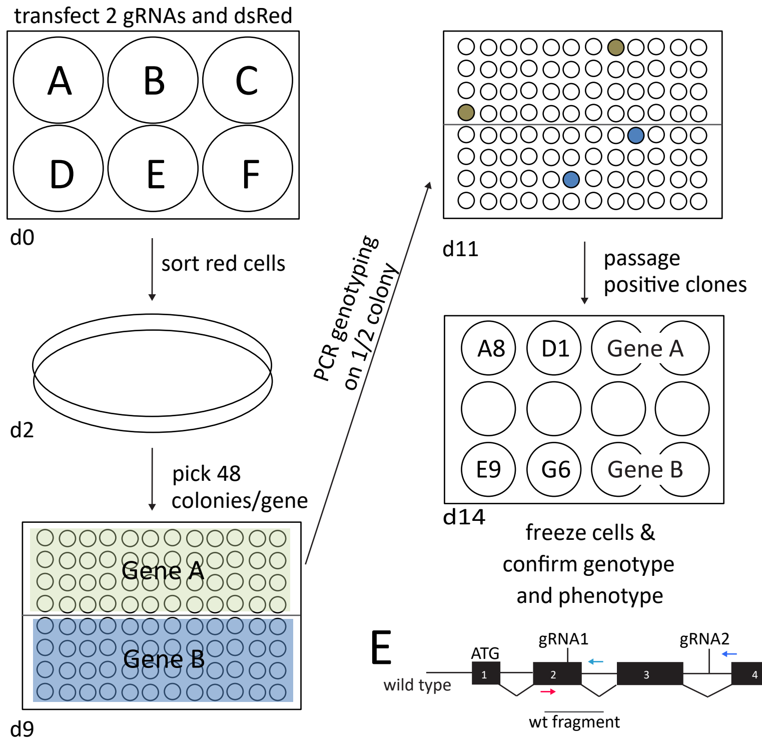

E

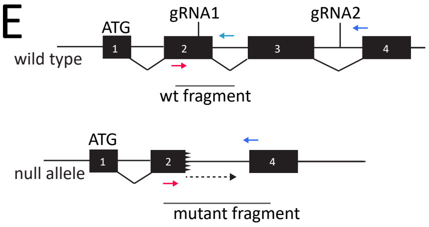

B

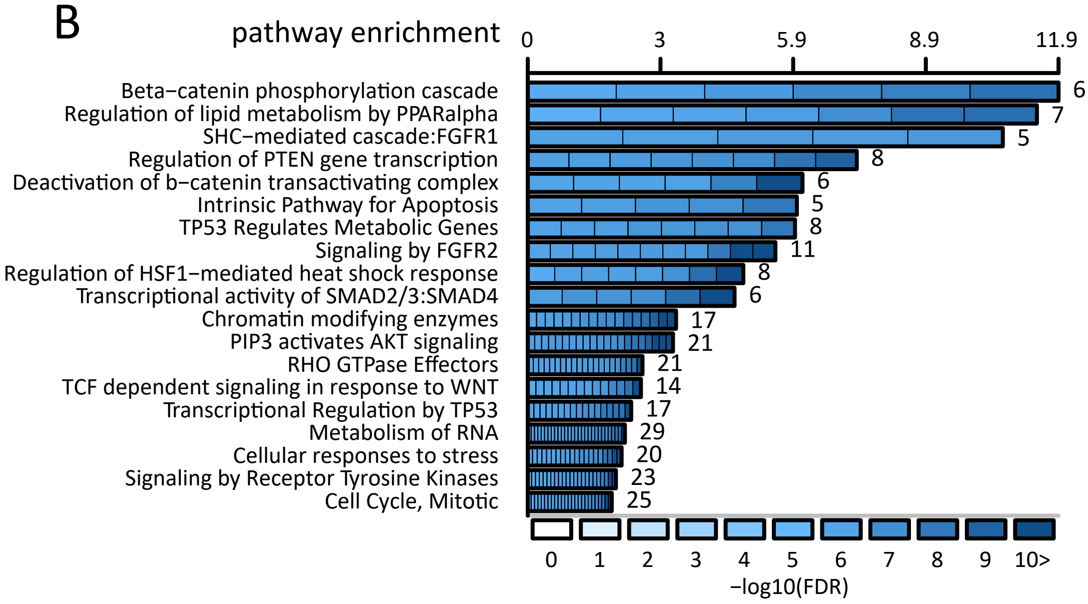

C

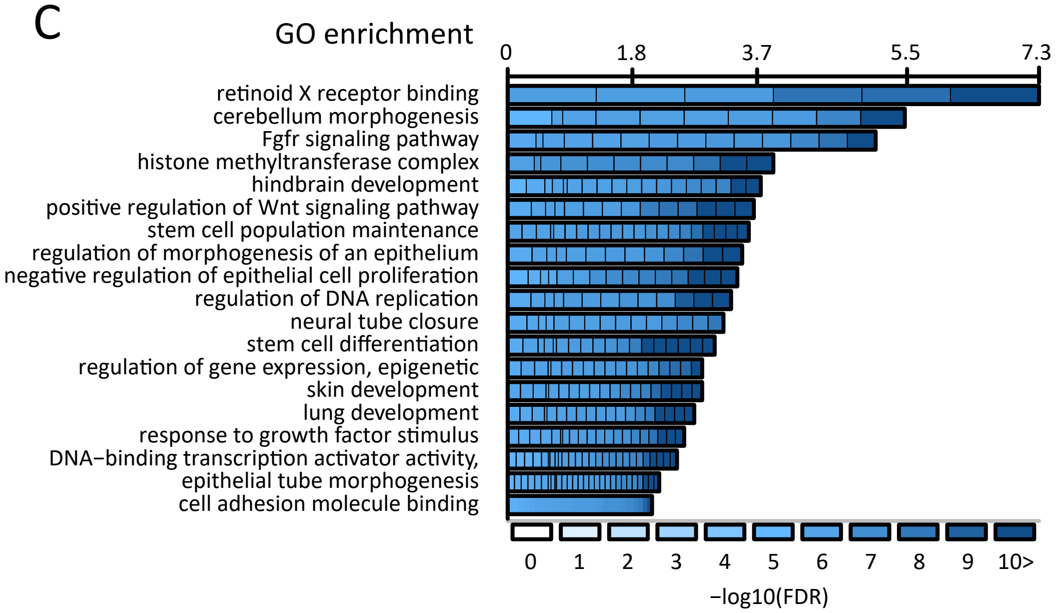

Appendix Figure S1

Appendix Figure S1

A The top 15 candidate genes from the haploid screen ordered by the number of screens (out of 35 screens) they were independently detected. The number of accumulated independent integration sites across all 35 screens and the calculated FDR are given for each hit (Dataset EV6).

B Significantly enriched pathways among screen hits, ranked by fold enrichment; colour code represents haploid screen-based FDR for genes in the GO and pathway categories. Numbers next to bars indicate the number of hit-genes within the category.

C Significantly enriched GO terms among screen hits as in **B**.

D Workflow to generate CRISPR/Cas9 knockouts in RC9 ESCs.

E Schematic strategy illustrating gRNAs and genotyping primers.

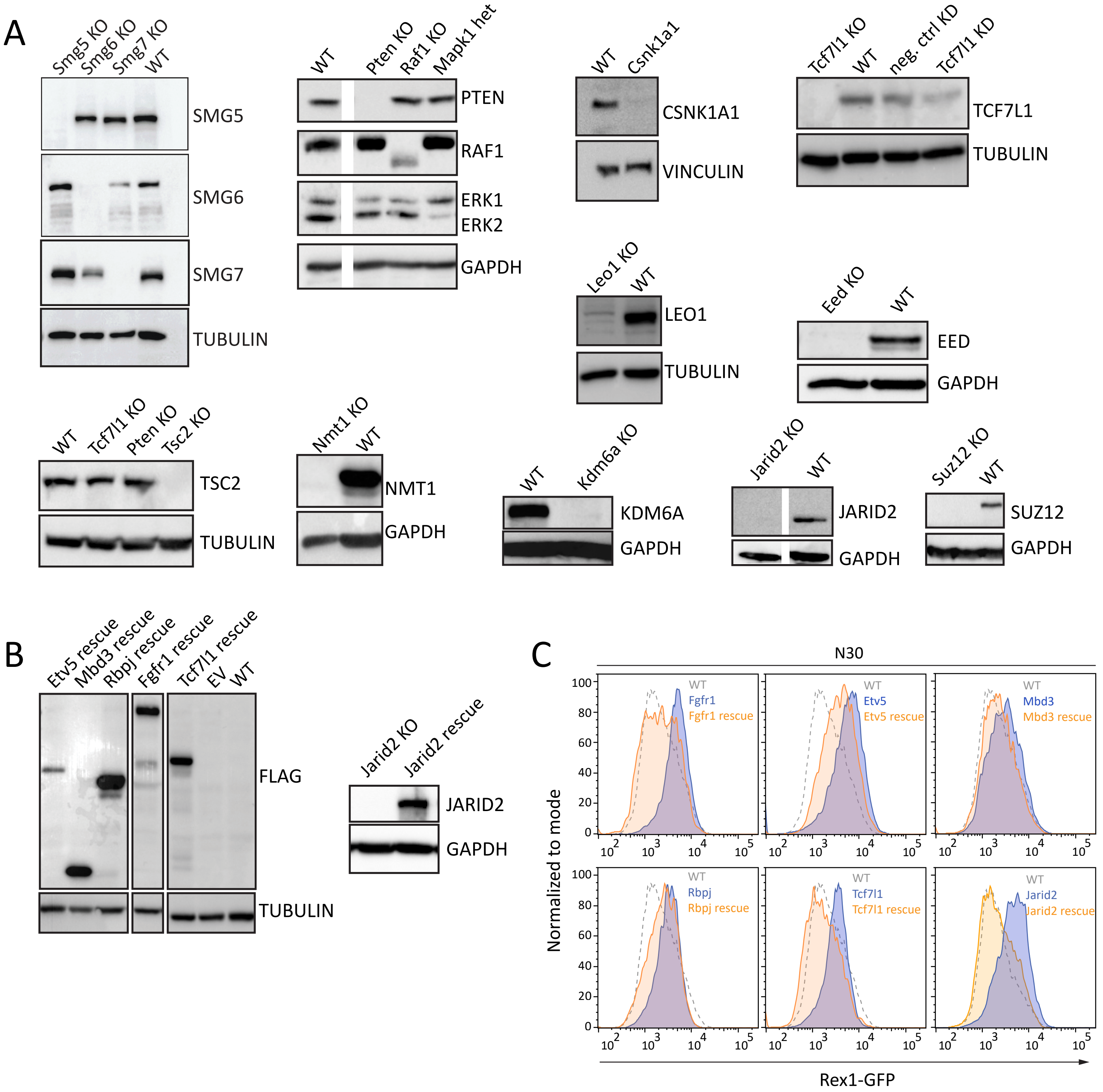

Appendix Figure S2

Appendix Figure S2

A Western analysis using indicated KOs and indicated antibodies. Tubulin, Gapdh or Vinculin were used as loading controls, as indicated.

B Anti-flag specific immunoblots for indicated KO^{rescue} ESCs upon forced expression of 3xflag rescue cDNAs from piggyBac-based transgenes driven from a CAG promoter (left panel). Jarid2 specific immunoblot to detect rescue-construct expression in Jarid2KO ESCs (right panel)

C Rex1-GFP levels measured by FACS showing restoration of differentiation behaviour in the indicated rescue cell lines at N30.

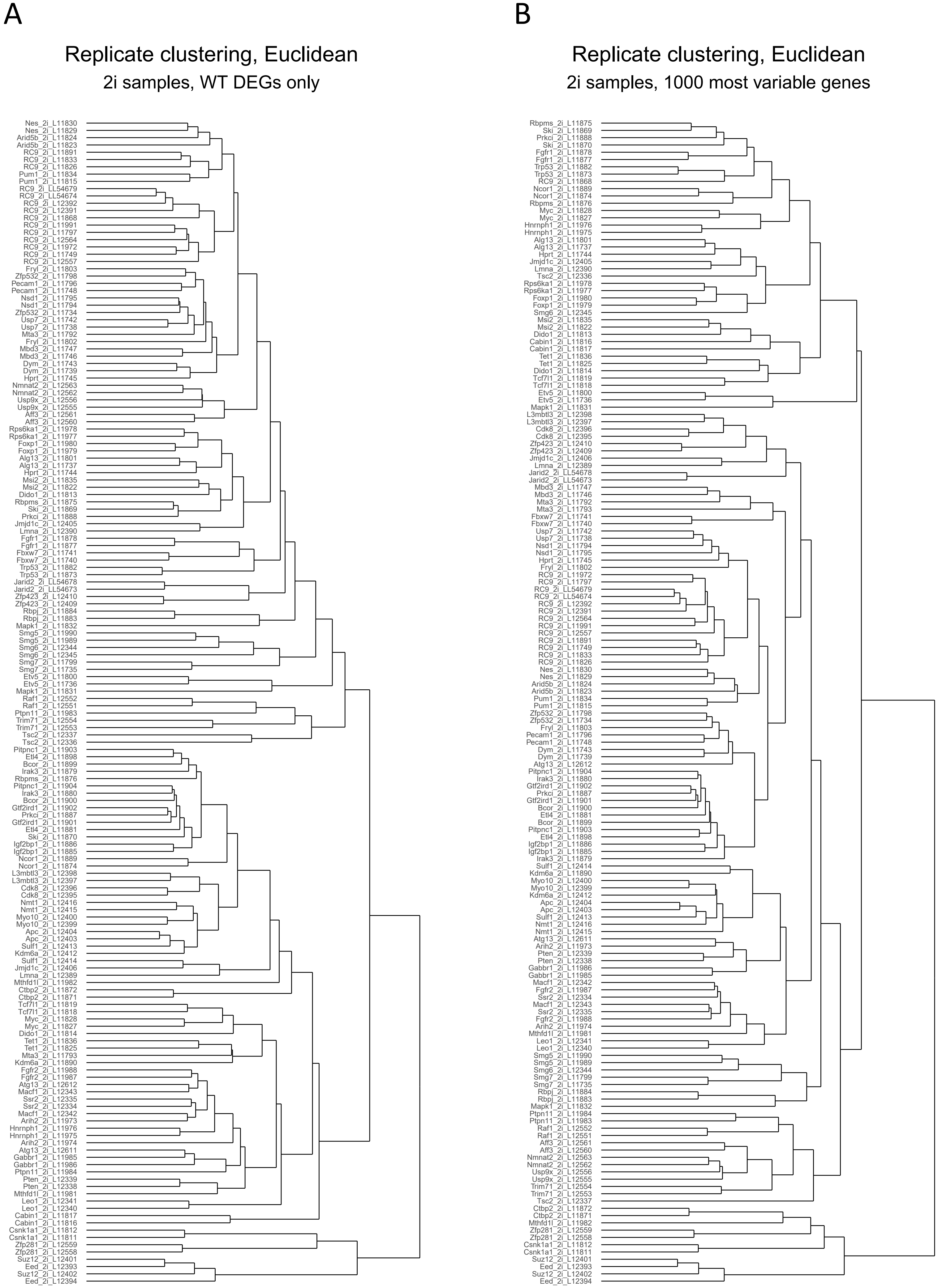

Appendix Figure S3

Appendix Figure S3

Dendrograms showing replicate clustering of KO and WT transcriptome profiles based on all DEGs in WT differentiation (**A**) or the 1000 top variant genes (**B**).

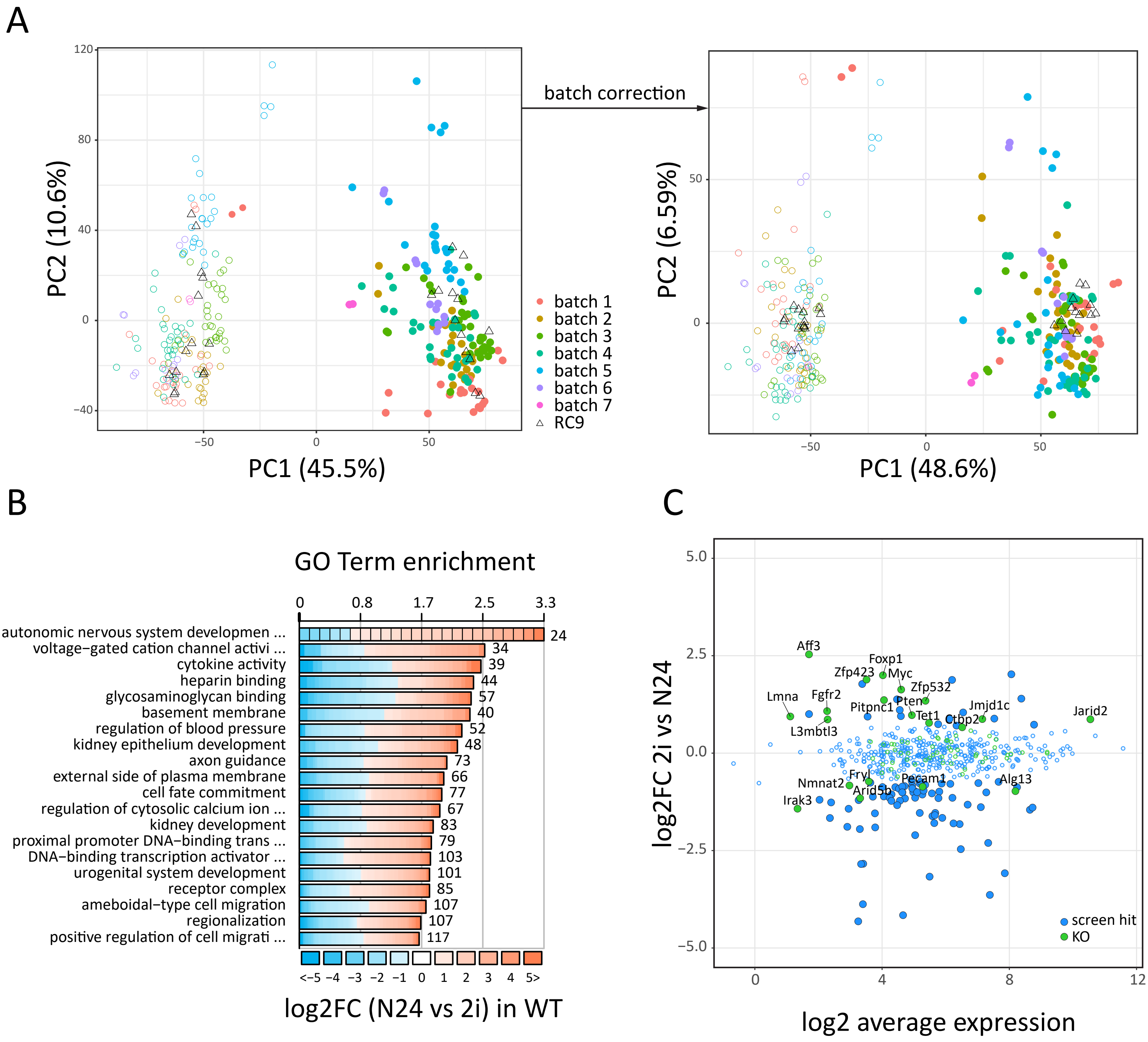

Appendix Figure S4

Appendix Figure S4

A PCA visualization of RNA-seq profiles in 2i and at N24 before (left) and after (right) batch correction. Open circles indicate samples in 2i, closed circles samples at N24. Black triangles show WT cells.

B GO term enrichment analysis of differentially expressed genes between 2i and N24 in WT ESCs ($FDR \leq 0.05$, H0: $|log2FC| < log2(1.5)$). The colour code indicates the log2FC in WT differentiation (N24 vs 2i) of individual genes in the GO-terms.

C Differential expression of genes at N24 vs. 2i in WT RC9 cells. Only Haploid screen hits (blue dots) and the 73 KO-genes are shown (green dots). Genes showing significant differential expression (DEGs) are shown as filled circles. All other genes as unfilled circles.

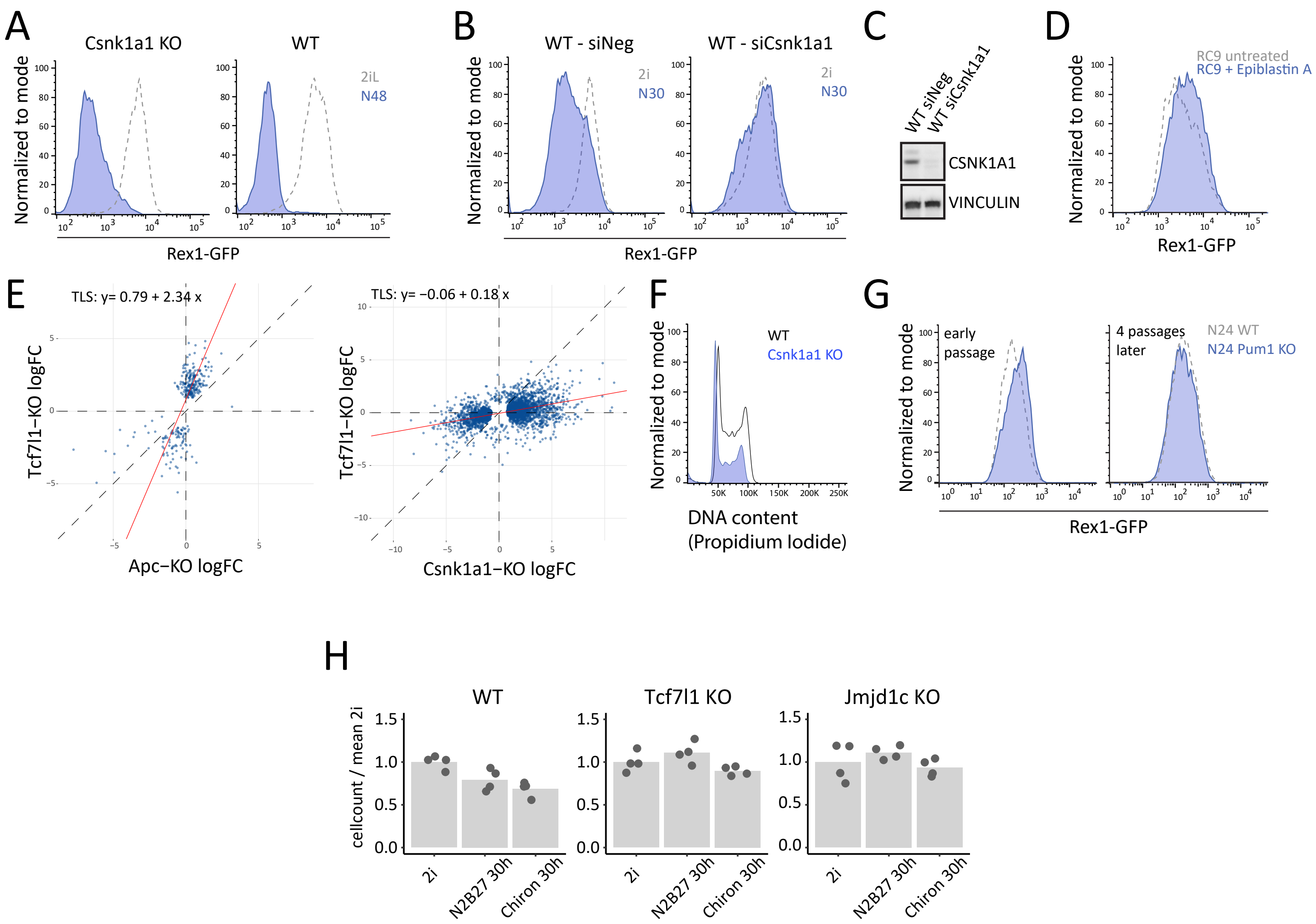

Appendix Figure S5

Appendix Figure S5

A Rex1-GFP FACS in *Csnk1a1* KO cells compared to WT at N48.

B Differentiation delay of Csnk1a1 siRNA knockdown cells compared to negative control siRNA knockdown at N30 measured by Rex1-GFP FACS.

C Reduction of protein after siRNA mediated knockdown of Csnk1a1 determined by immunoblotting.

D Rex1-GFP levels measured by FACS at N24 showing the effect of Epiblastin A addition during differentiation.

E Dot-plot showing regression analysis of KO-induced changes at N24, comparing *Tcf7l1* KO to *Apc* and *Csnk1a1* KOs. Total least squares (TLS) analysis results are indicated. All genes that show significant expression changes (FDR ≤ 0.05, H0: |log2FC| < log2(1.5)) in each of the KOs in each of the pairwise comparisons are plotted.

F Propidium Iodide staining and FACS analysis illustrating the proliferation defect of Csnk1a1 KO compared to WT cells in N2B27 based medium.

G Rex1-GFP analysis at N24 of *Pum1* KO cells, showing a relatively strong differentiation defect at early passages which is lost in later passages.

H Quantification of cell number in WT and KO lines after 30h in N2B27 or N2B27 supplemented with Chiron 99021. 2i samples serve as control. Measurements are normalized to the mean of 2i samples (n=4). Each dot corresponds to one measurement.

A

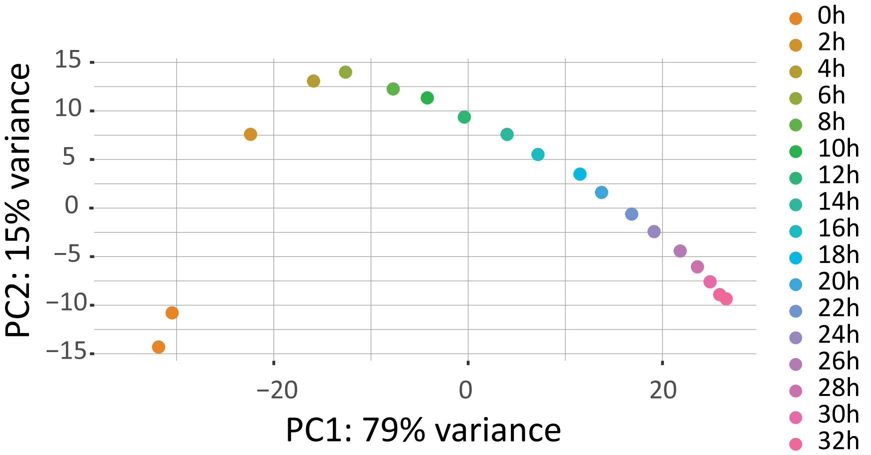

B

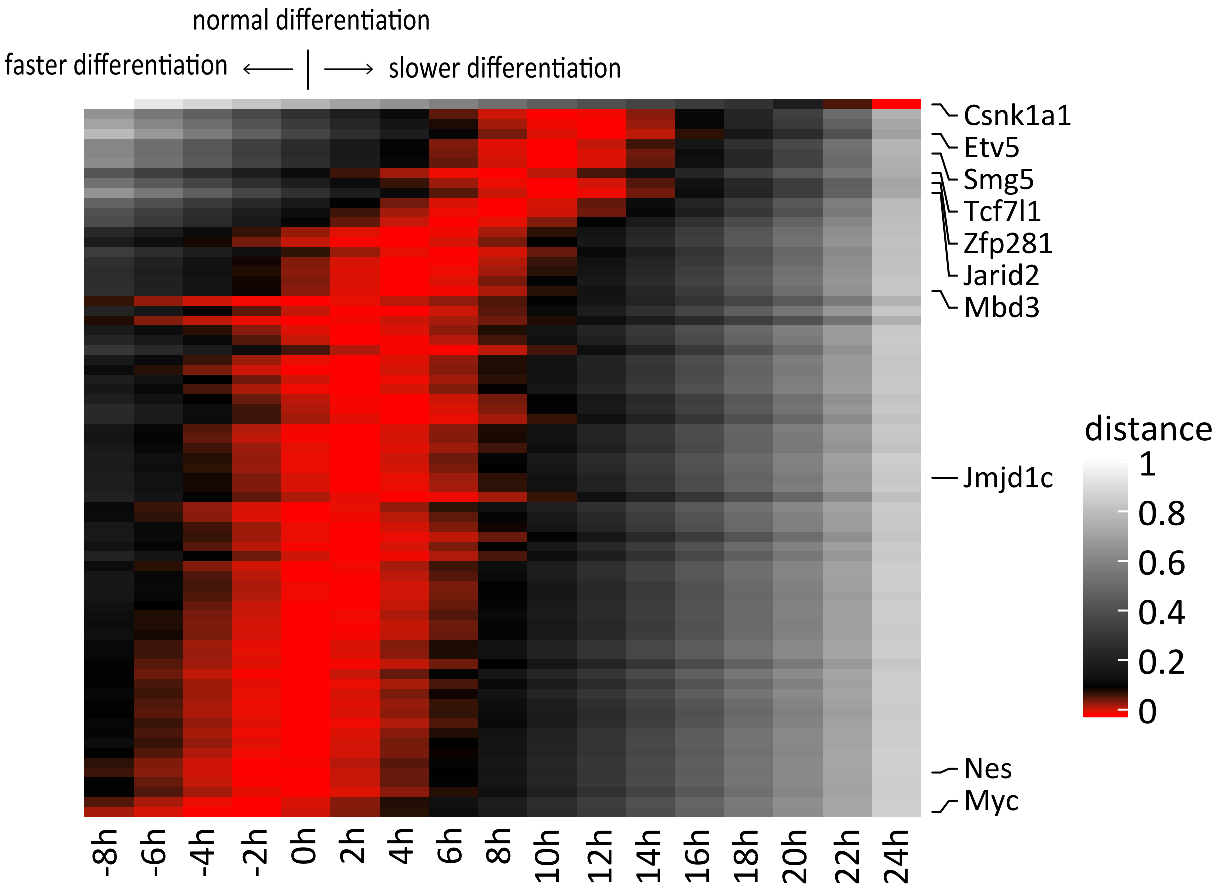

Appendix Figure S6

Appendix Figure S6

A Principle component analysis (PCA) of the 2h resolved WT differentiation time course sampled by RNA-seq. 0h corresponds to cells in 2i.

B Heatmap showing differentiation delays of the 73 KO lines quantified using the expression of all 3068 genes differentially expressed in WT between 2i and N24. Red bars indicate the closest correlation to that specific time-point. Positive values indicate delayed differentiation; negative values more rapid differentiation compared to WT. Each line corresponds to one KO. Selected KOs are annotated. (see also EV Dataset 2)

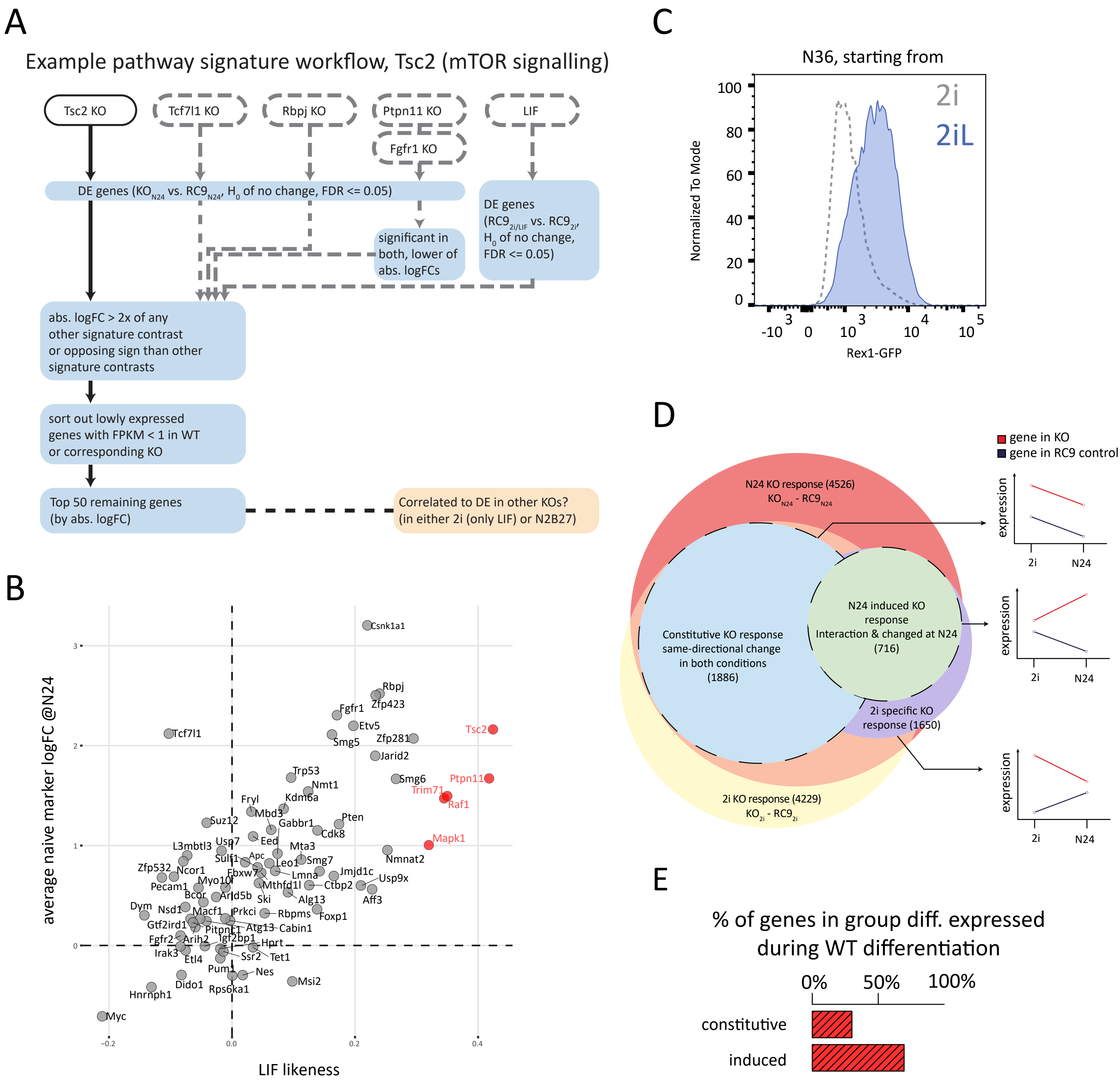

Appendix Figure S7

Appendix Figure S7

A Schematized computational strategy to derive non-overlapping gene-expression footprints for the five signalling pathways.

B Average log2FC in naive marker expression induced by KOs at N24 (y-axis) and their 'LIF-likeness' in 2i (x-axis) to indicate correlation of expression of LIF regulated genes with the differentiation strength in all KOs. Overall correlation is r=0.65. The KOs showing the strongest correlations (*Tsc2*, *Ptpn11, Raf1, Mapk1* and *Trim71* KOs) are indicated in red.

C Flow analysis showing delayed Rex1-GFP downregulation in cells starting from cultures in 2i/LIF compared to cells starting from 2i.

D Euler diagram showing the overlap of defined gene groups for determination of the constitutive and the N24 induced KO-response. Panels on the right show exemplary gene expression behaviours. The constitutive and the N24 induced KO response group of genes were used for further analysis.

E Percentages of genes in the N24 induced KO response (N24 induced) or constitutive KO response (constitutive) clusters that significantly change during WT differentiation (FDR ≤ 0.05, H0: $|\log2FC| < \log2(1.5)$).

Appendix Figure S8

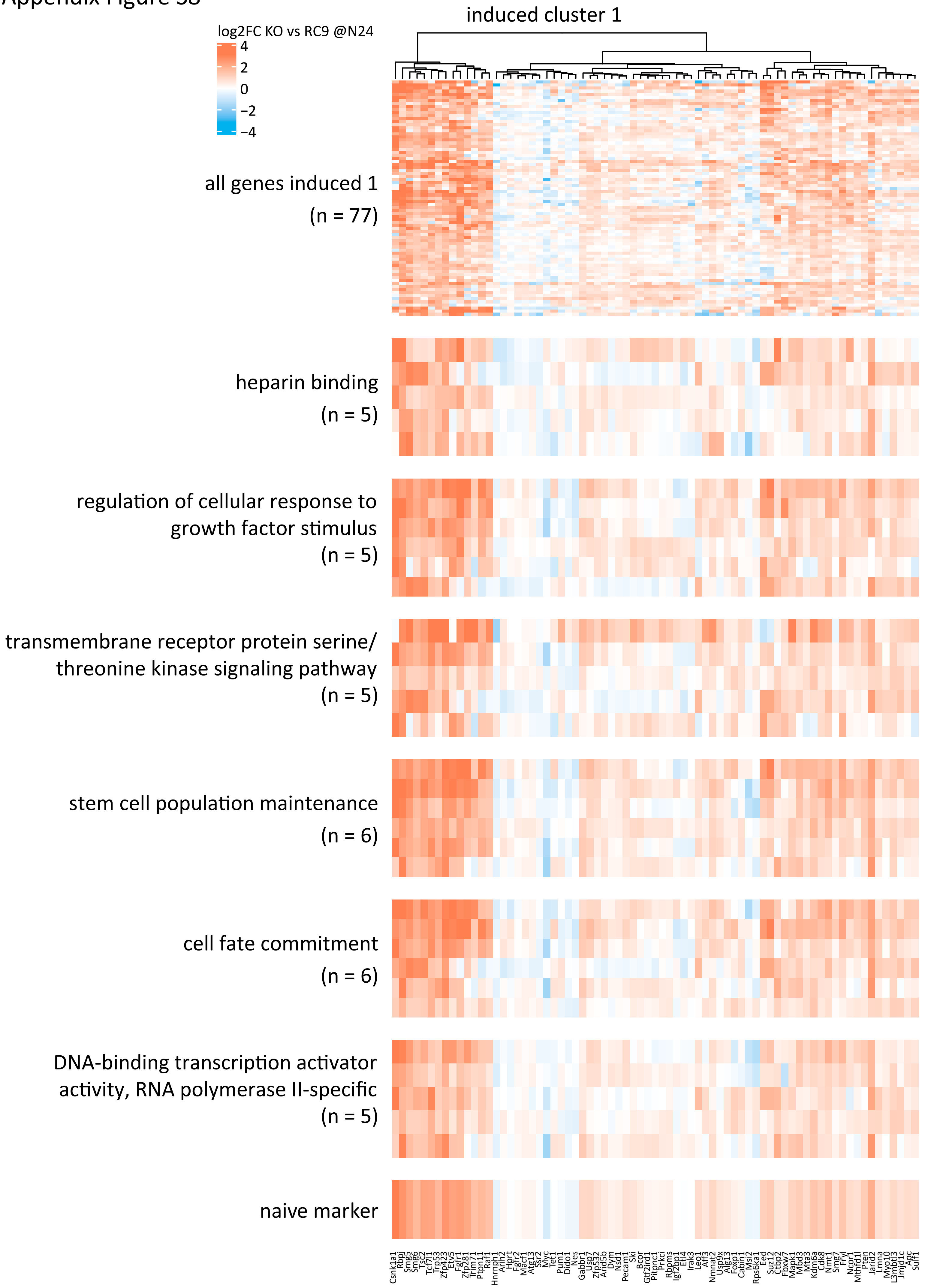

Appendix Figure S9

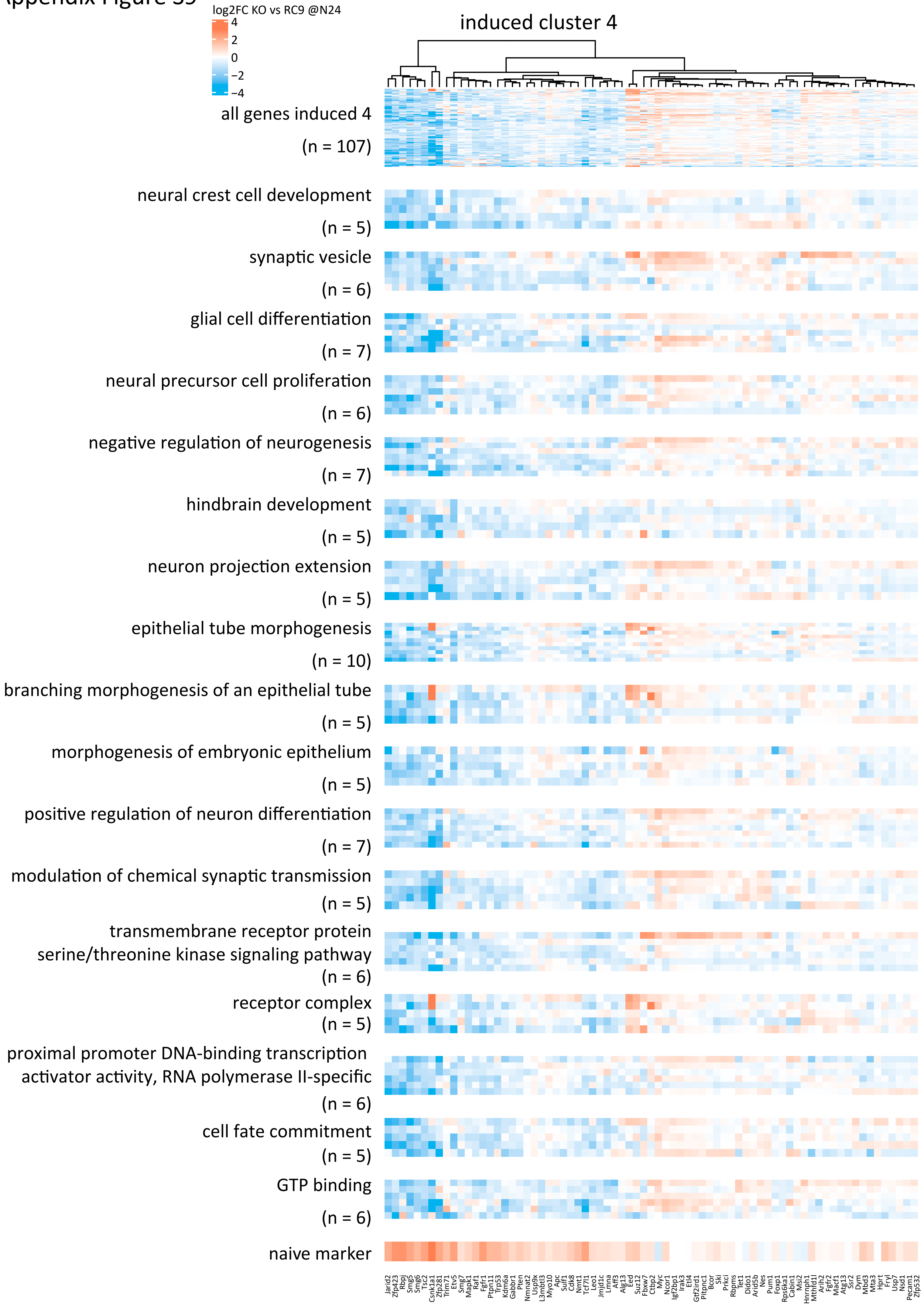

Appendix Figure S10

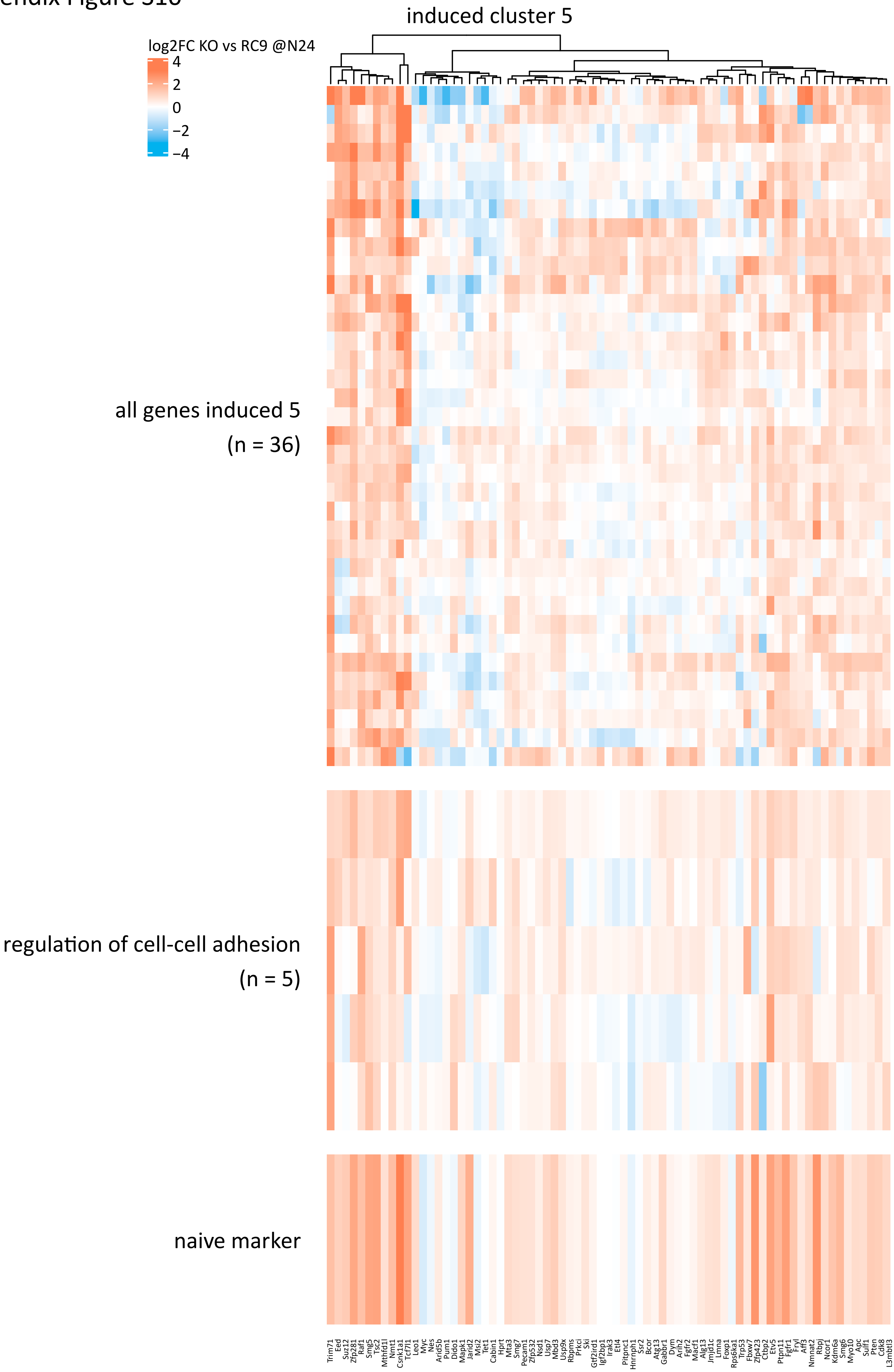

Appendix Figure S11

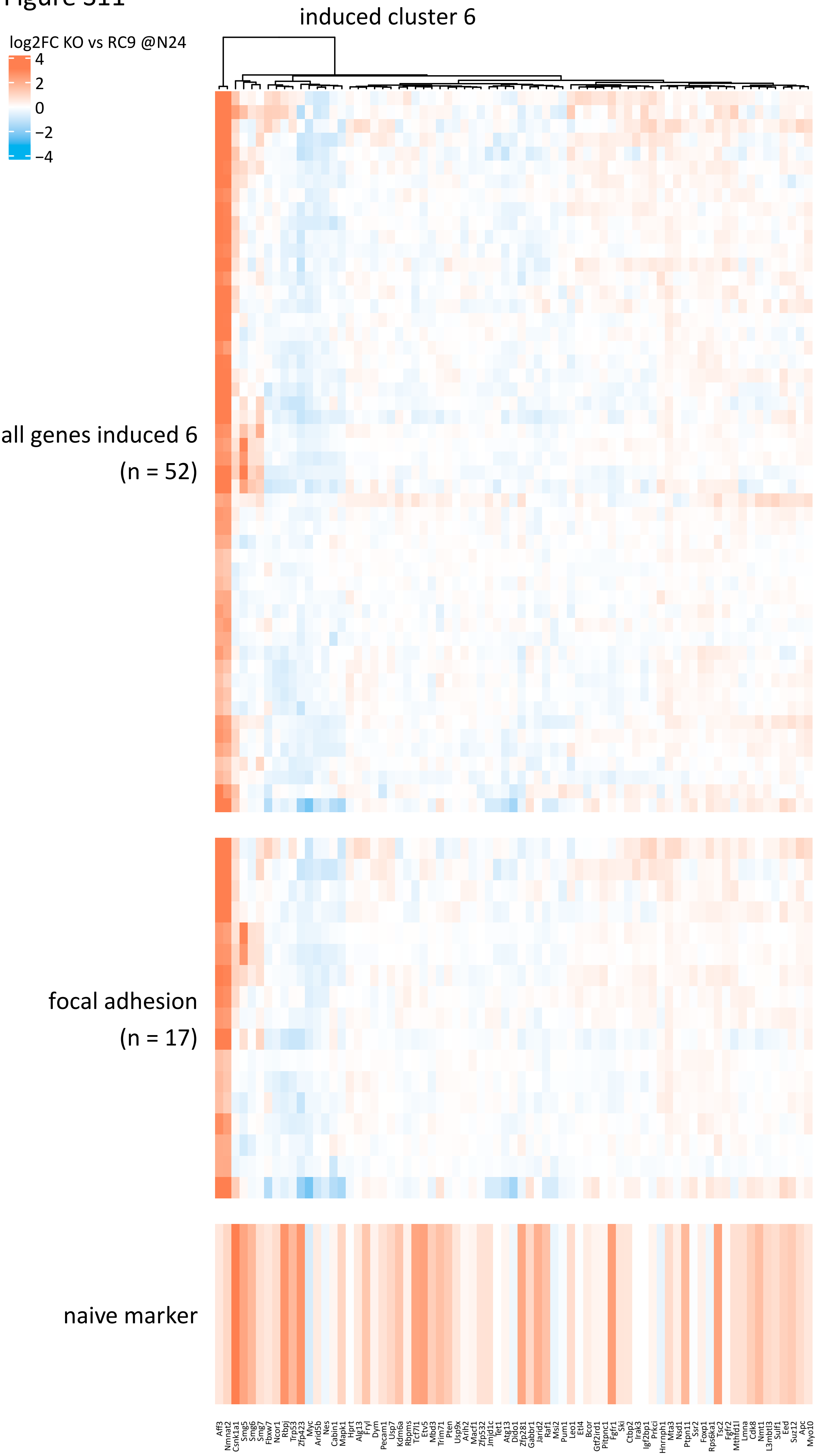

Appendix Figure S12

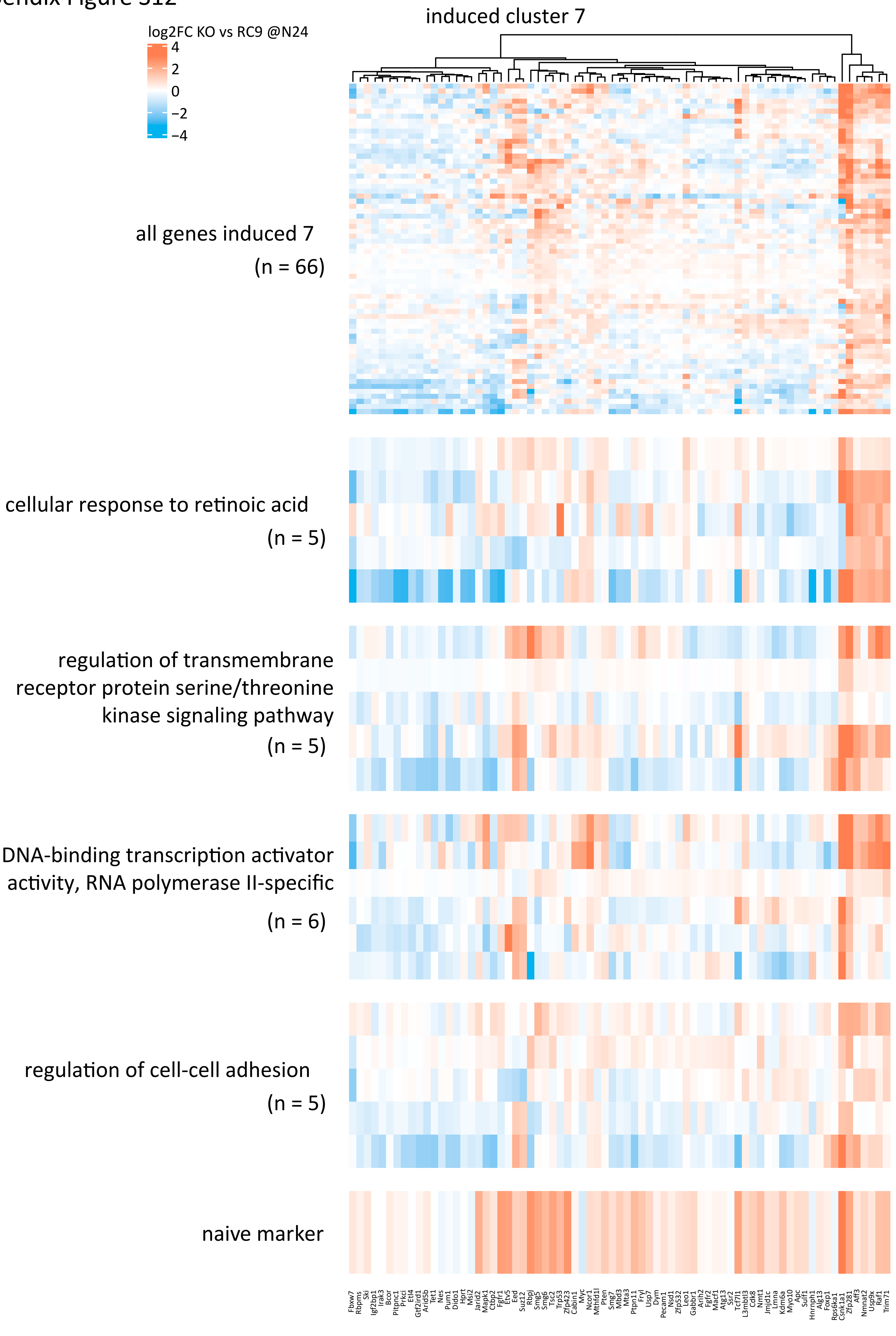

Appendix Figure S13

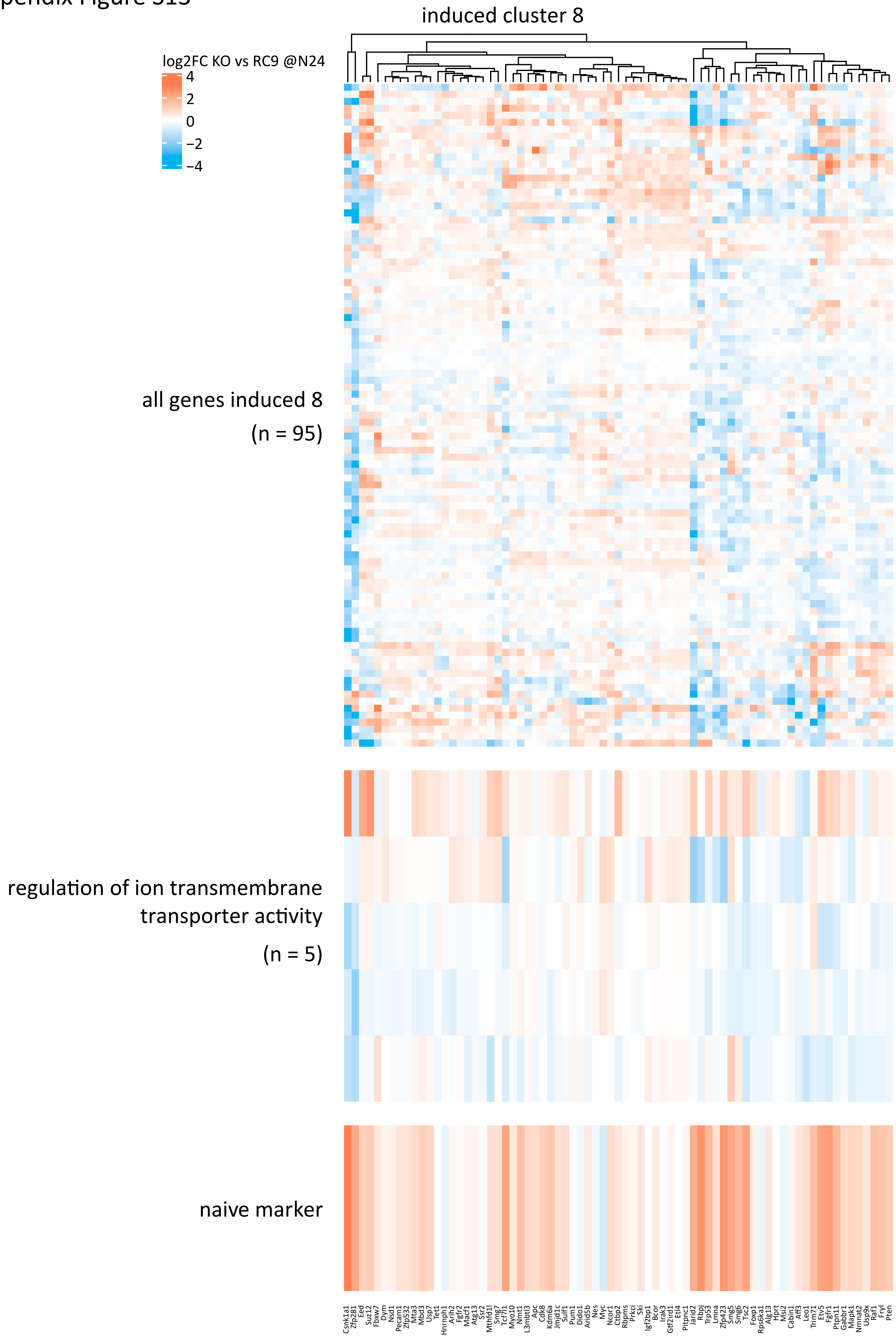

Appendix Figure S14

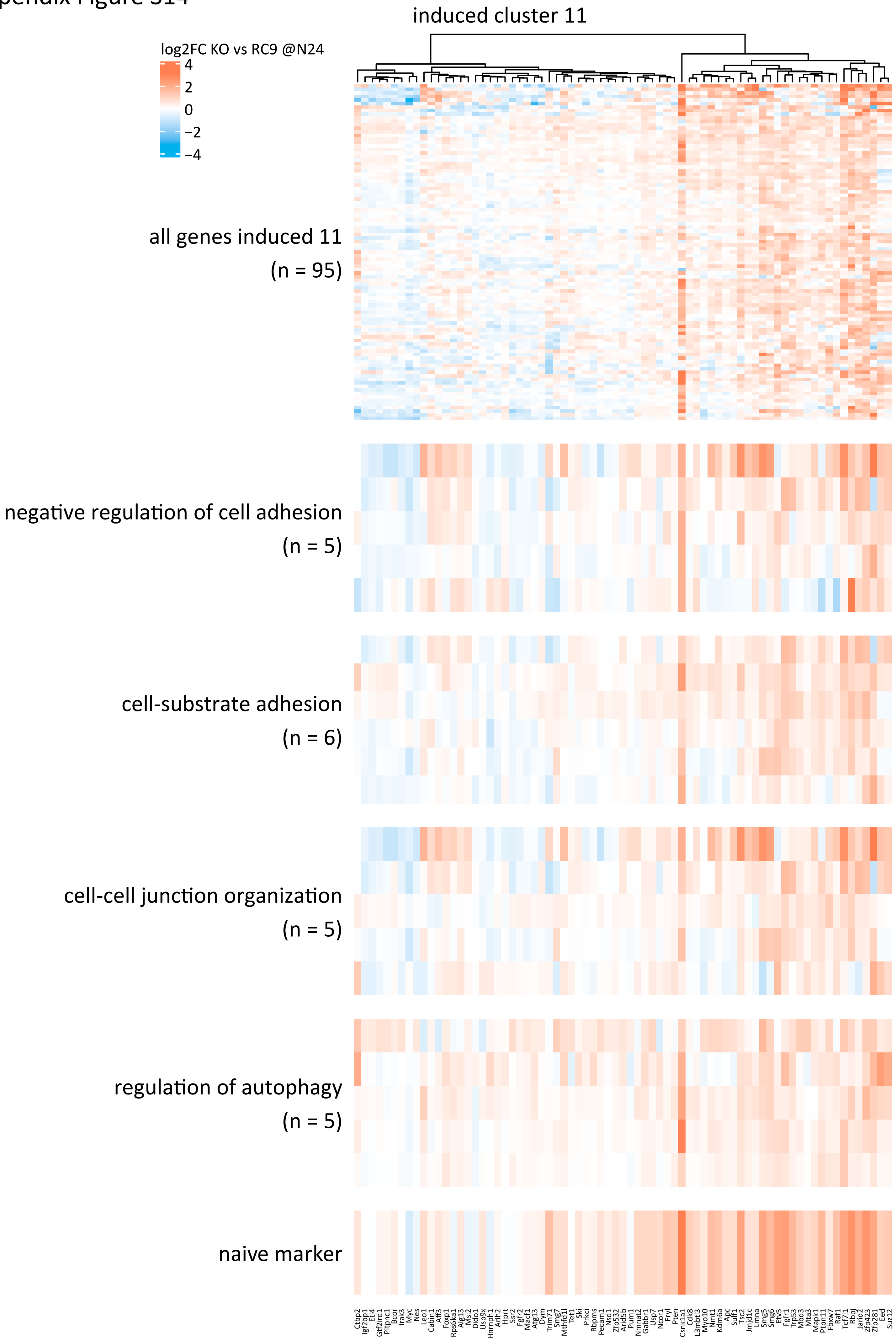

Appendix Figure S15

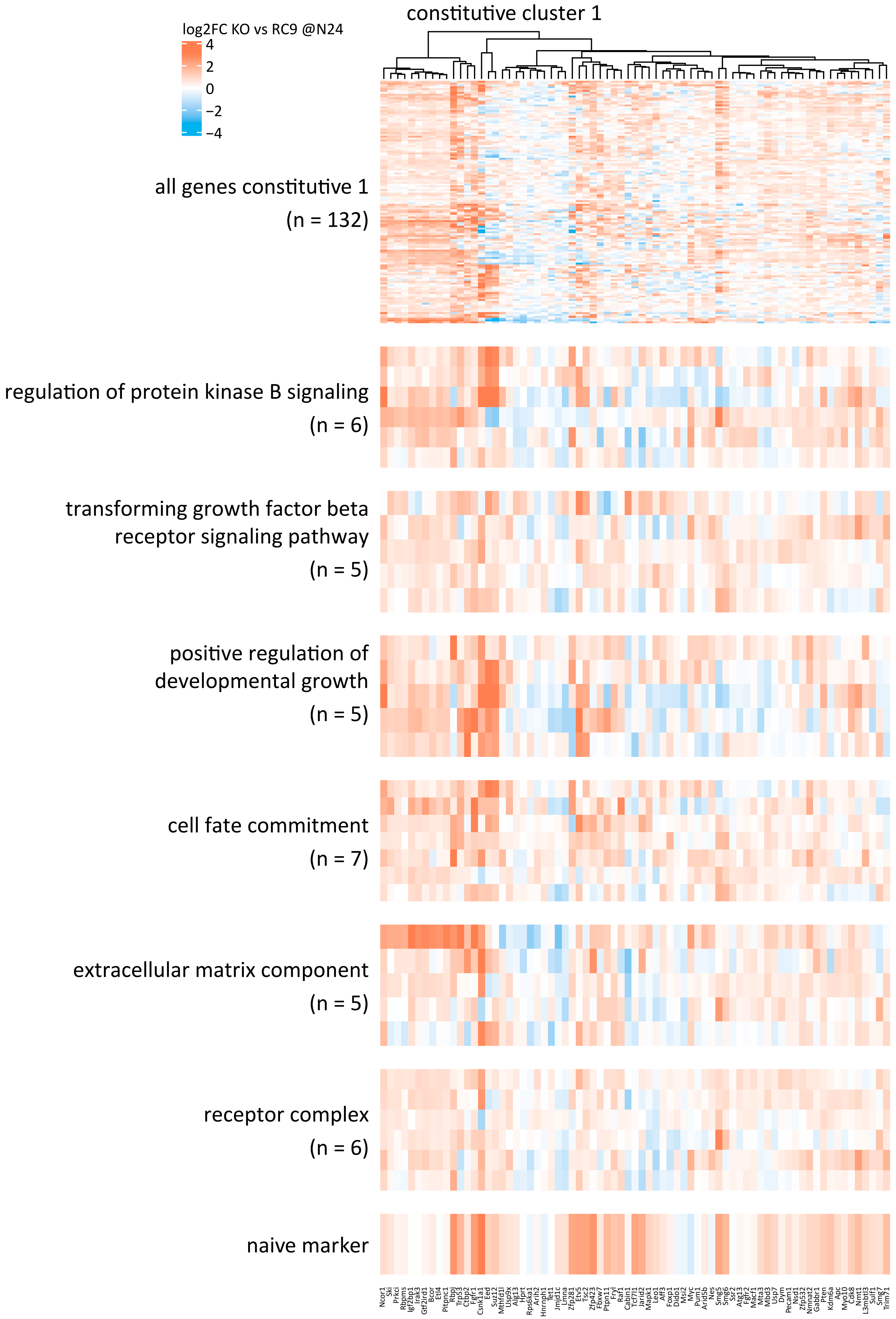

Appendix Figure S16

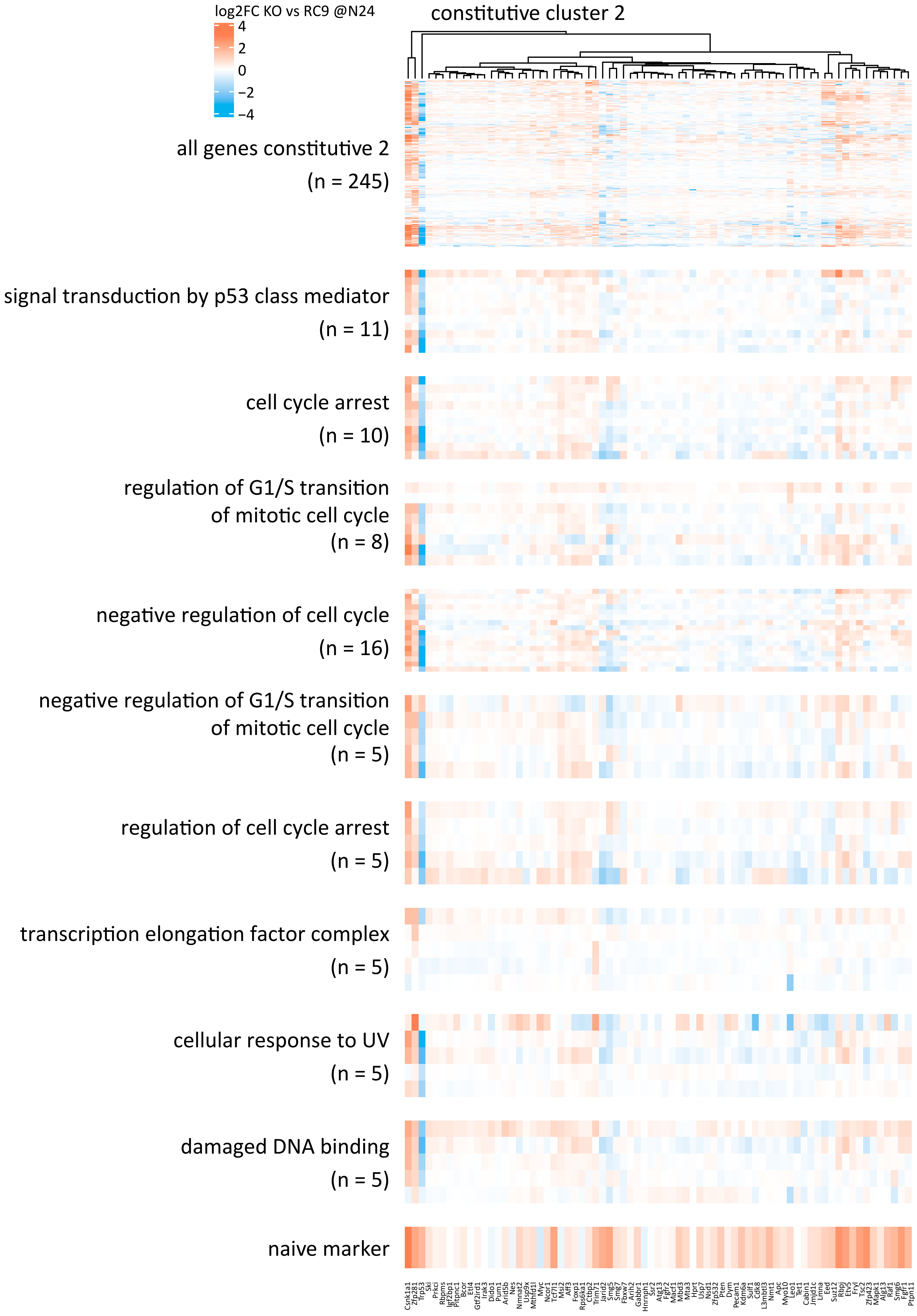

Appendix Figure S17

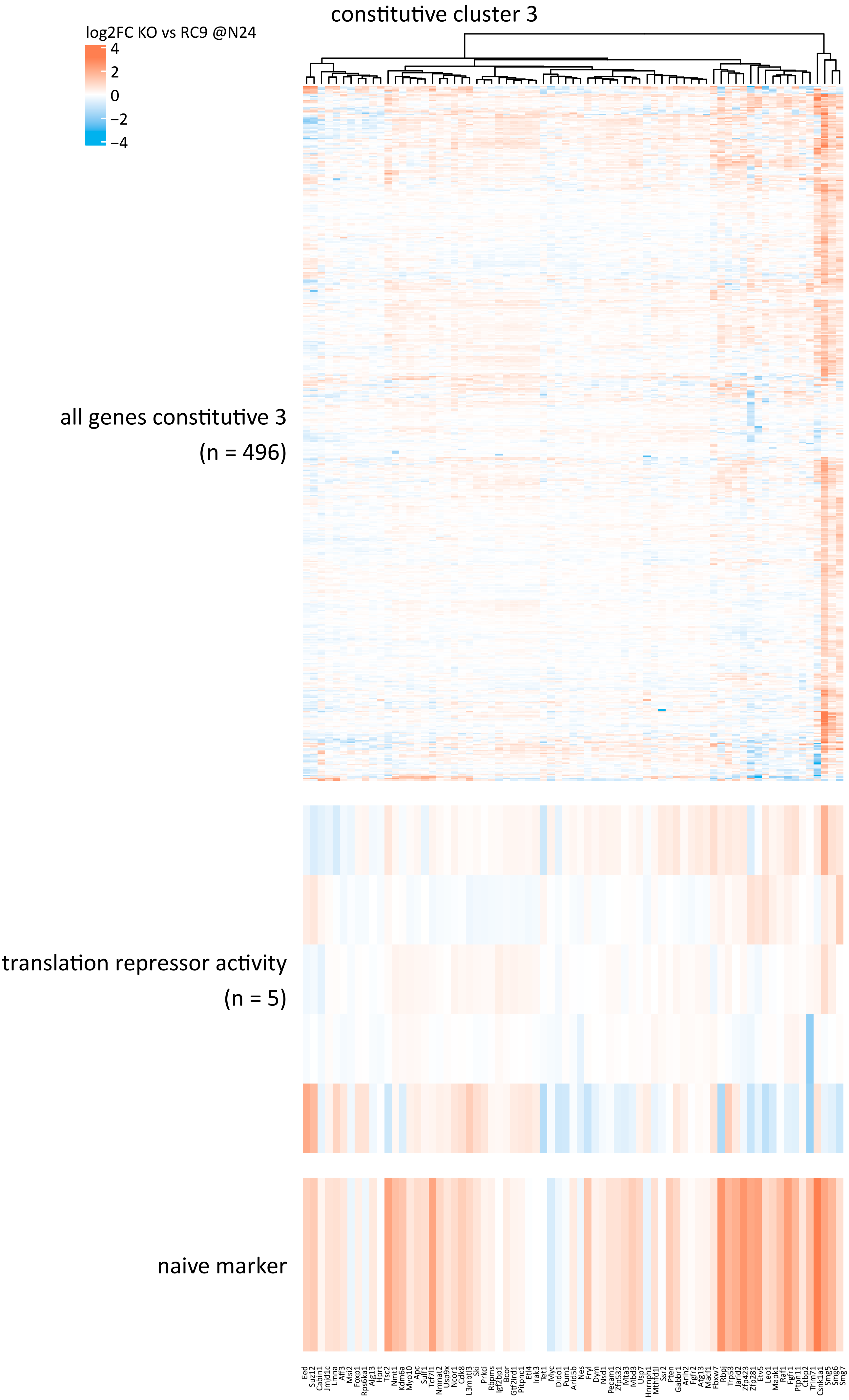

Appendix Figure S18

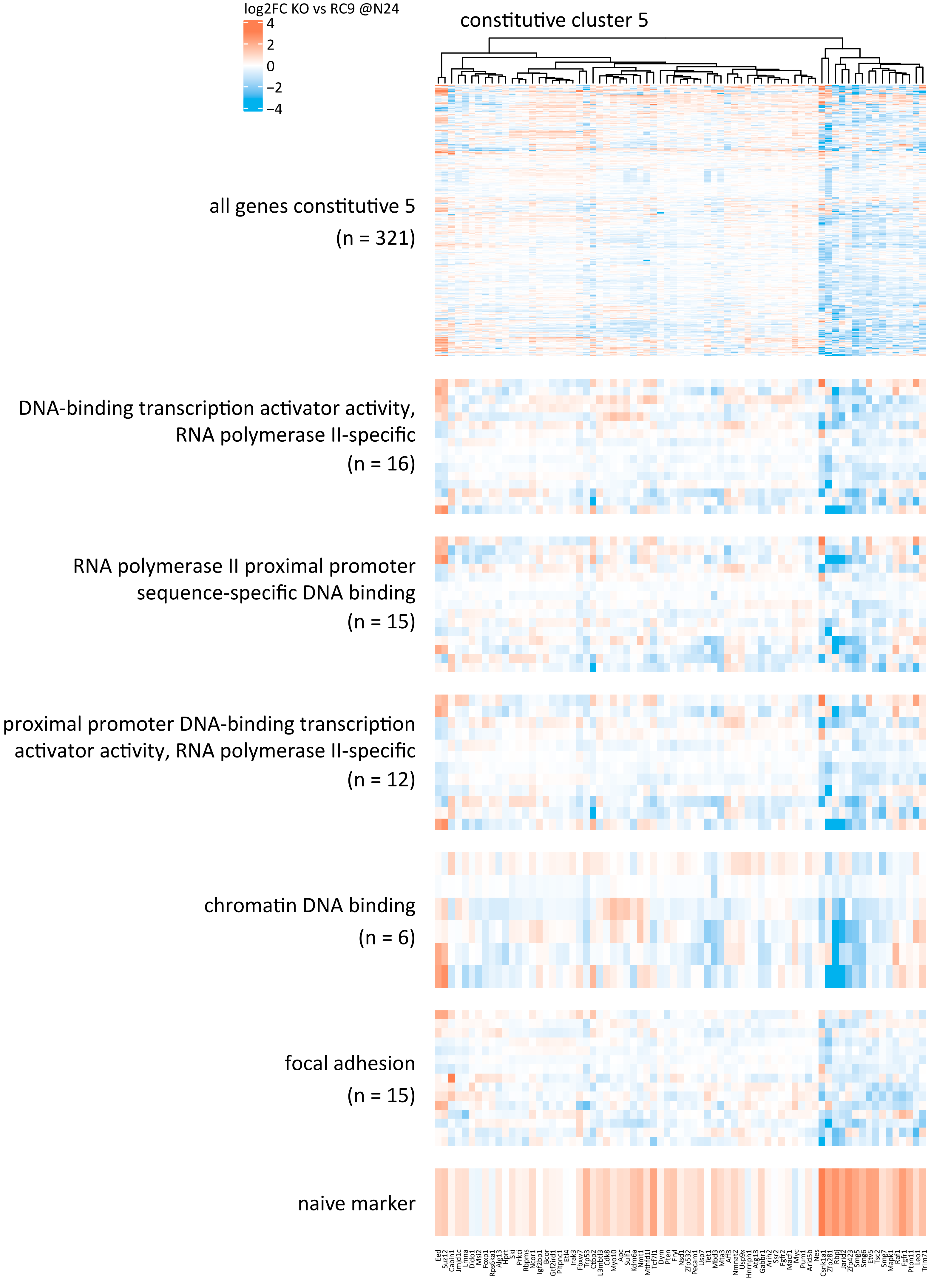

Appendix Figure S19

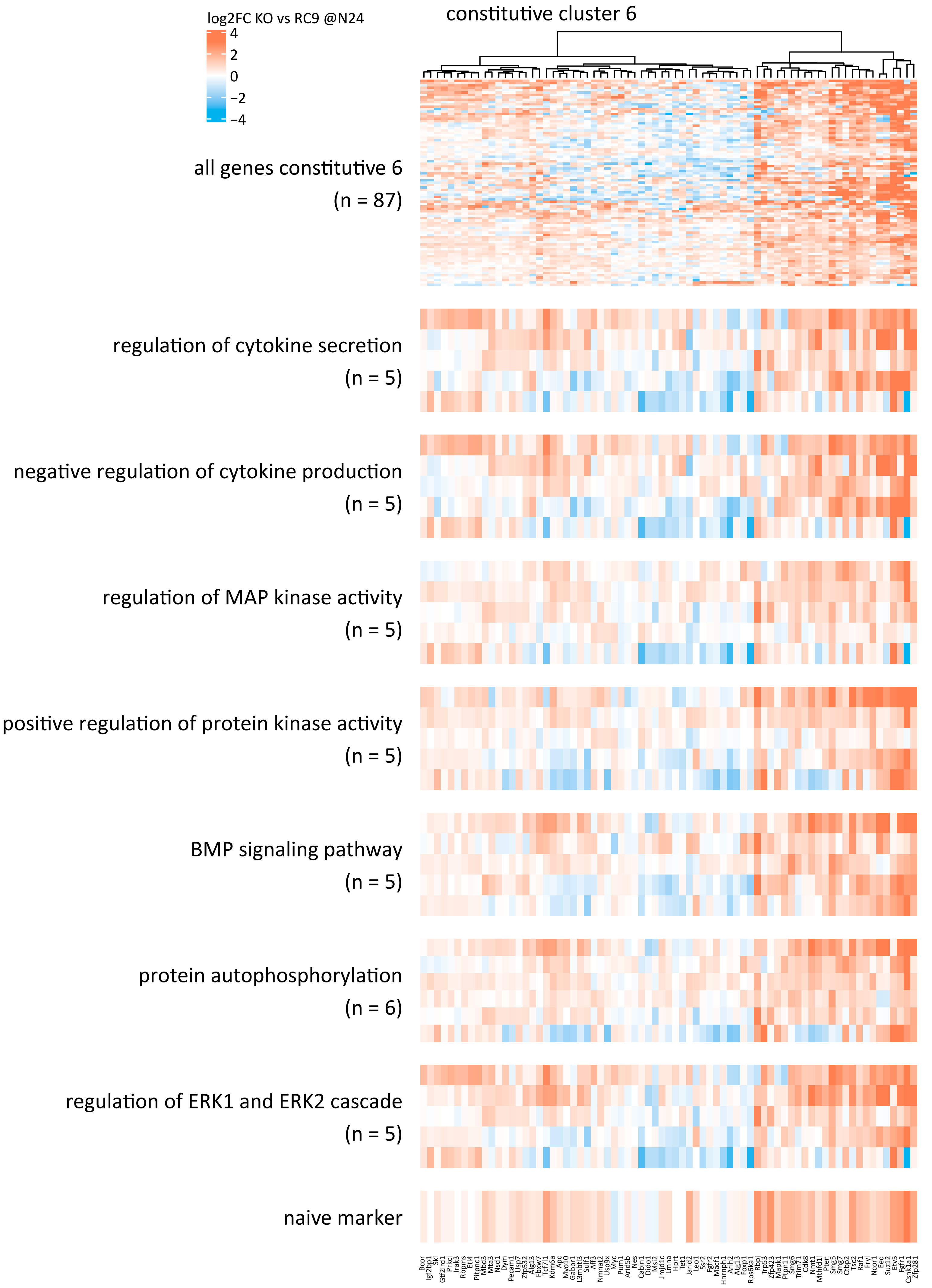

Appendix Figure S20

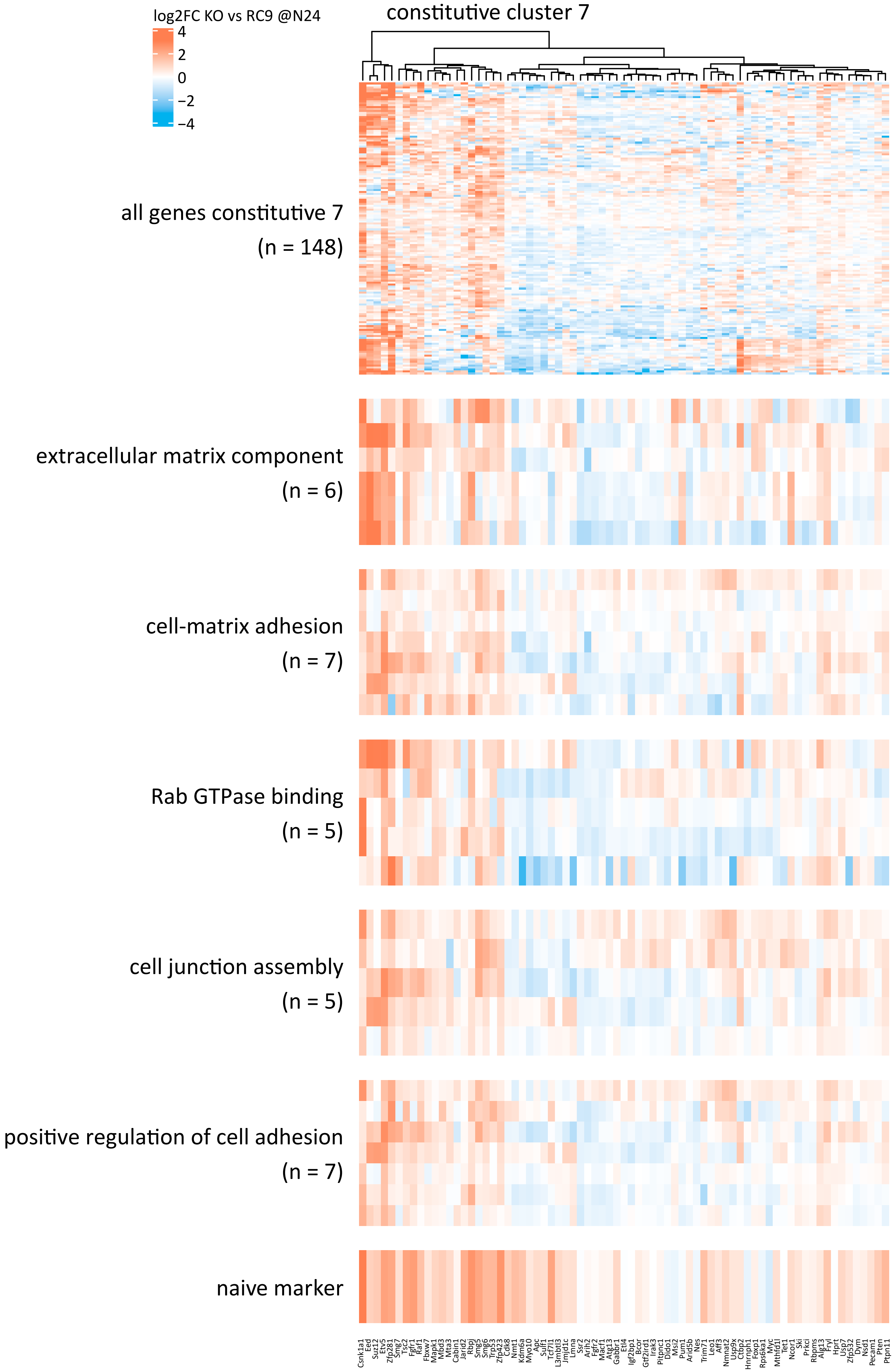

Appendix Figure S8 – S20

Heatmaps showing hierarchical clustering of selected constitutive and N24 induced clusters and subsets of cluster genes identified in GO term analysis in all KOs at N24. Upper panels show heatmaps of all genes in the selected cluster. Middle panels show genes included in selected GO terms to determine clusters of KO regulating a specific GO term and agreement of cluster and GO term expression. Bottom panel shows average naïve marker log2FC (to identify potential phenotype dependence of clustering).
