# Supplementary material for: Cooperative genetic networks drive embryonic stem cell transition from naïve to formative pluripotency
Source: EMBO J. 2021 Mar 9;40(8):e105776. doi: 10.15252/embj.2020105776 (PMC8047444; doi:10.15252/embj.2020105776)
Supplement: Supplementary file 3 — Expanded View Figures PDF [file EMBJ-40-e105776-s005.pdf]

Expanded View Figures

Figure EV1. Systematic transcriptional profiling of a mutant ESC library.

A RNA-seq-derived fold changes relative to WT of indicated naïve marker genes in indicated KOs in 2i (colour scale shows FDR, only genes with FDR $\leq$ 0.05 are shown).
B RNA-seq-derived fold changes relative to WT of indicated formative marker genes in indicated KOs in 2i (colour scale shows FDR, only genes with FDR $\leq$ 0.05 are shown).
C Rex1-GFP FACS analysis of WT cells in 2i, at N24 and at N30, transfected with negative control or *Klf2*-specific siRNAs.
D Comparison of the number of differentially expressed genes at N24 (FDR $\leq$ 0.05, H0: $|log2FC| < log2(1.5)$) to the average naïve marker log2FC at N24 (phenotype strength).
E Correlation of *Tcf7l1* KOs to *Jmjd1c* KOs regarding log2FCs at N24 between knockout and WT control. Each dot corresponds to one gene. Only genes showing significance (FDR $\leq$ 0.05, H0: $|log2FC| < log2(1.5)$) in either one of the two KOs are plotted. Red line: total least square regression; regression coefficients are shown.

 © 2021 The Authors

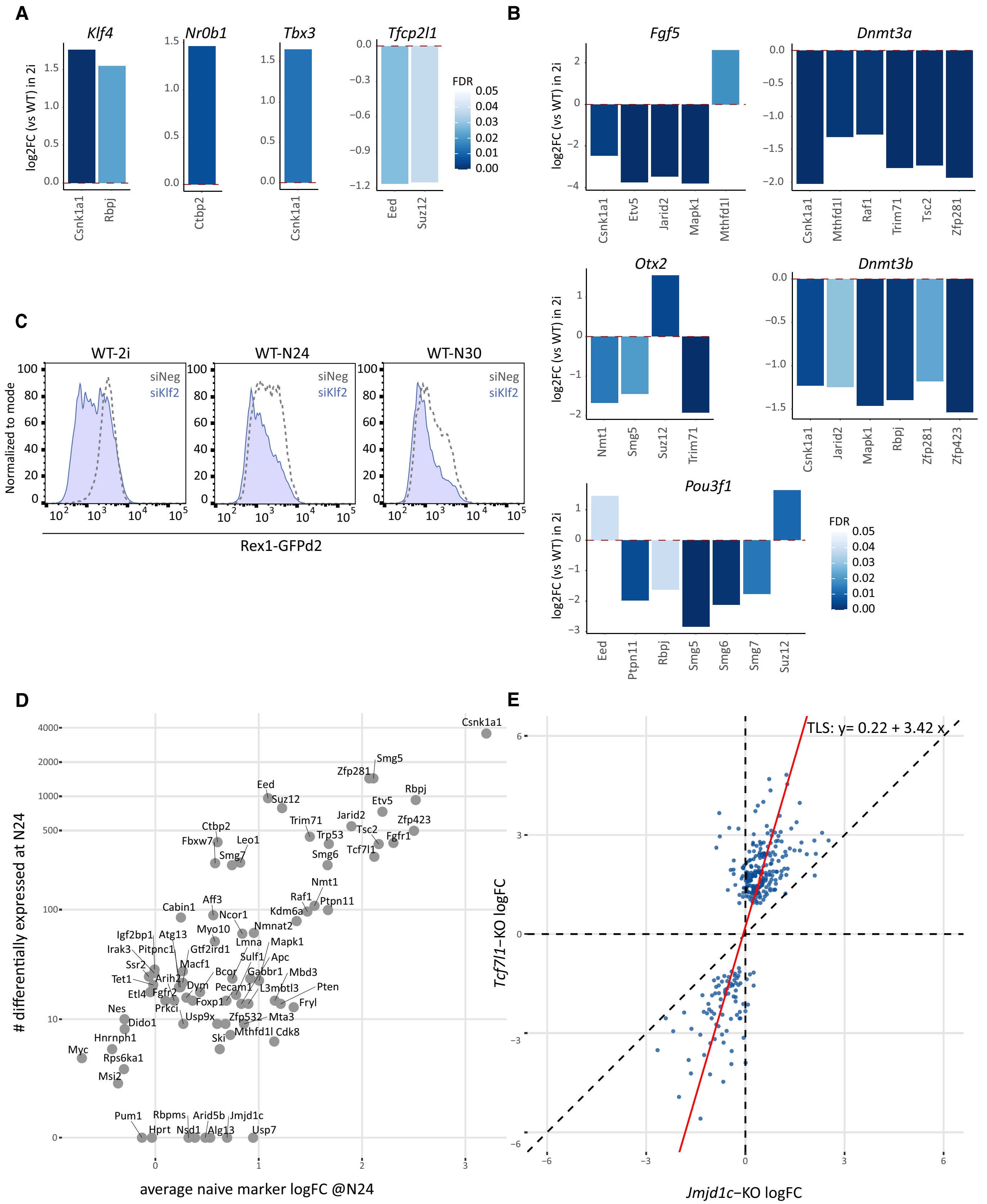

A
Klf4
Nr0b1
Tbx3
Tfcp2l1
log2FC (vs WT) in 2i
Csnk1a1
Rbpj
Ctbp2
Eed
Suz12
FDR
B
Fgf5
Dnmt3a
Otx2
Dnmt3b
Pou3f1
C
WT-2i
WT-N24
WT-N30
Normalized to mode
siNeg
siKlf2
Rex1-GFPd2
D
differentially expressed at N24
average naive marker logFC @N24
E
TLS: y= 0.22 + 3.42 x
Tcf7l1–KO logFC
Jmjd1c–KO logFC

Figure EV1.

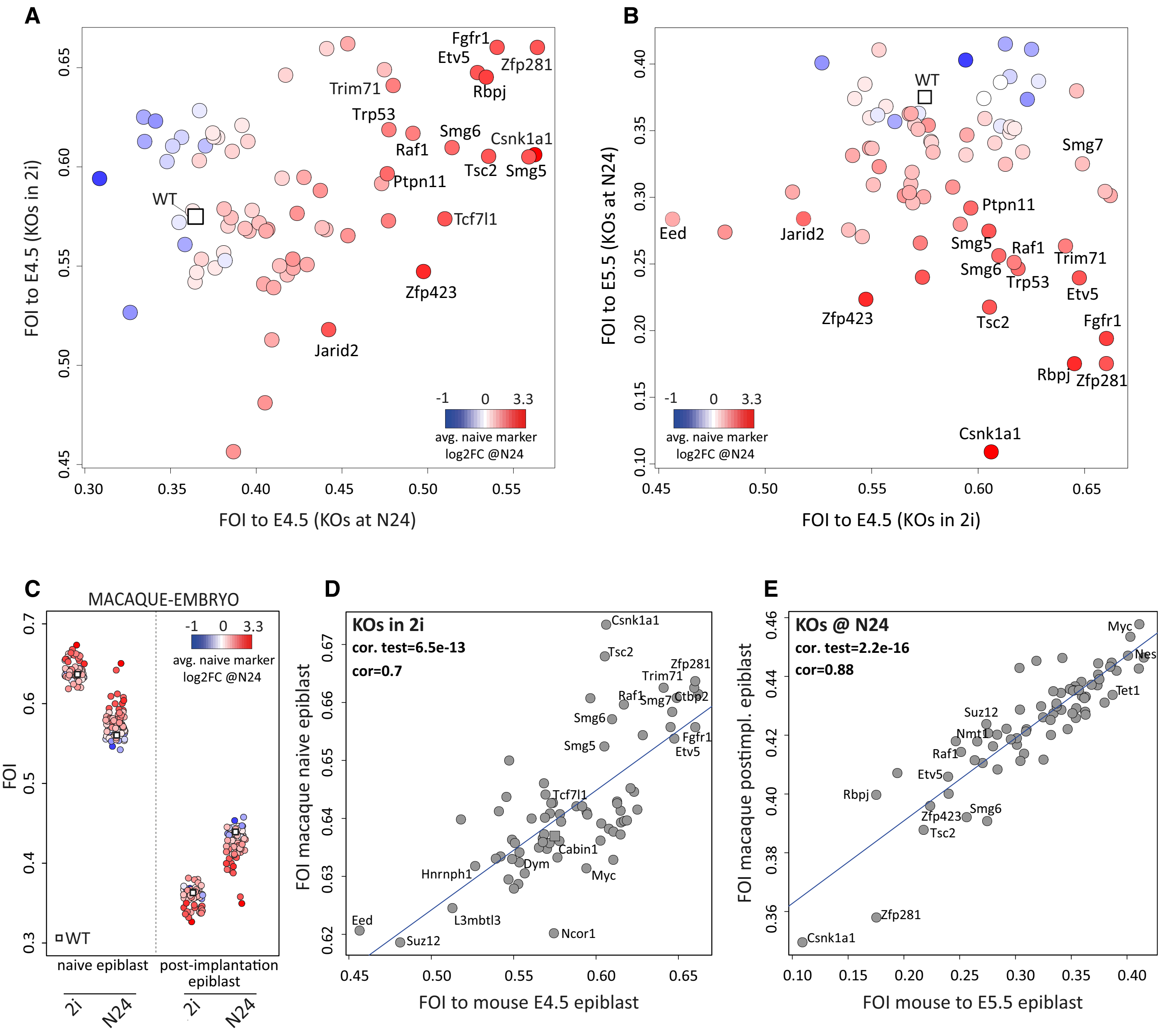

Figure EV2. Comparing KO expression profiles to the *in vivo* pre- to post-implantation epiblast transition.

A FOIs to the E4.5 epiblast of all KOs in 2i compared with FOI to the E4.5 epiblast of all KOs at N24. The average naïve marker log2FC in KOs at N24 is indicated as colour gradient (strength of differentiation phenotype).

B FOIs of 2i samples to E4.5 epiblast (x-axis) and FOIs of N24 samples to E5.5 epiblast (y-axis). The average naïve marker log2FC in KOs at N24 is indicated as colour gradient.

C FOIs between 2i and N24 expression data and macaque naïve and post-implantation epiblast, computed using all expressed genes (log2FPKM > 0) for all KOs. Each dot represents one KO. Average naïve marker log2FC in KOs at N24 is indicated by a colour gradient.

D Correlation plot of FOIs between 2i profiles and mouse naïve (E4.5) epiblast and FOIs between 2i profiles and macaque naïve epiblast (correlation significance is indicated in the plot).

E Correlation plot of FOIs between N24 profiles and mouse E5.5 epiblast and FOIs between N24 profiles and macaque post-implantation epiblast (significance of correlation is indicated in the plot).

 © 2021 The Authors

Figure EV3. Identification of an extended naïve pluripotency network.

A Comparison of the strength of the linkage to the naïve network (naïve association, R^2 values; x-axis) to the log2FC of all genes during the naïve to formative transition in WT (y-axis). upNAGs are indicated in red, downNAGs in blue.

B *Klf2* expression at the indicated stages. Expression levels (FPKM) in compacted morula (cMOR), expanded ICM (eICM), E4.5 epiblast (EPI) are shown derived from single-cell RNA-seq data sets from human, marmoset and mouse pre-implantation development. Box-plots were generated utilising the GRAPPA web app (Boroviak *et al*, 2018) and indicate the first and third quartile of expression levels. The midline identifies the median. Each dot corresponds to an individual cell.

C Plots showing expression of *Pdgfa* (top) and its cognate receptor *Pdgfra* (bottom) during mouse pre-implantation embryo development in a white (low) to orange (high) gradient.

D Principal component analysis of published single-cell RNA-seq data sets showing that Dimension 3 (Dim 3) separates pre- from post-implantation epiblast *in vivo* (magnification of plot in Fig 5A).

© 2021 The Authors

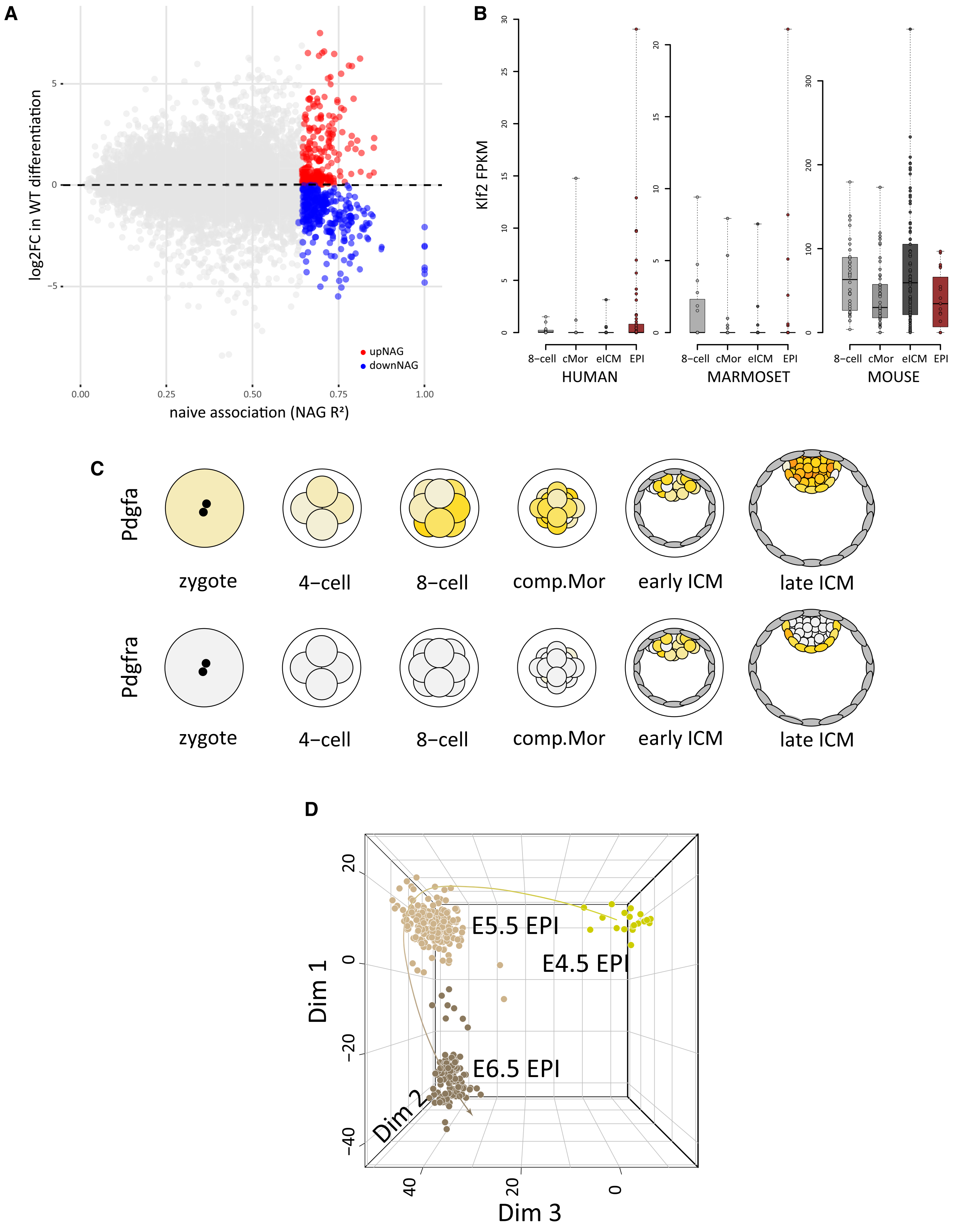

Figure EV3.

 © 2021 The Authors

Figure EV4. Signalling pathways and gene expression modules regulated by multiple KO genes.

A Heatmap showing average log2FCs (KO^{N24} versus RC9^{N24}) of constitutive KO response gene clusters (c1-c12). For reference, average naïve marker log2FCs versus WT at N24 are shown for each KO in a separate row below.

B As for (A) for N24-induced KO response gene clusters (i1-i12).

C Fisher test-based enrichments of ChIP targets, up/downNAGs and pathway-defining gene sets in constitutive clusters are indicated by circles. Size indicates log2 fold over-representation; the colour gradient encodes adjusted *P*-values.

D Similar to Fig EV4C for N24-induced clusters.

© 2021 The Authors

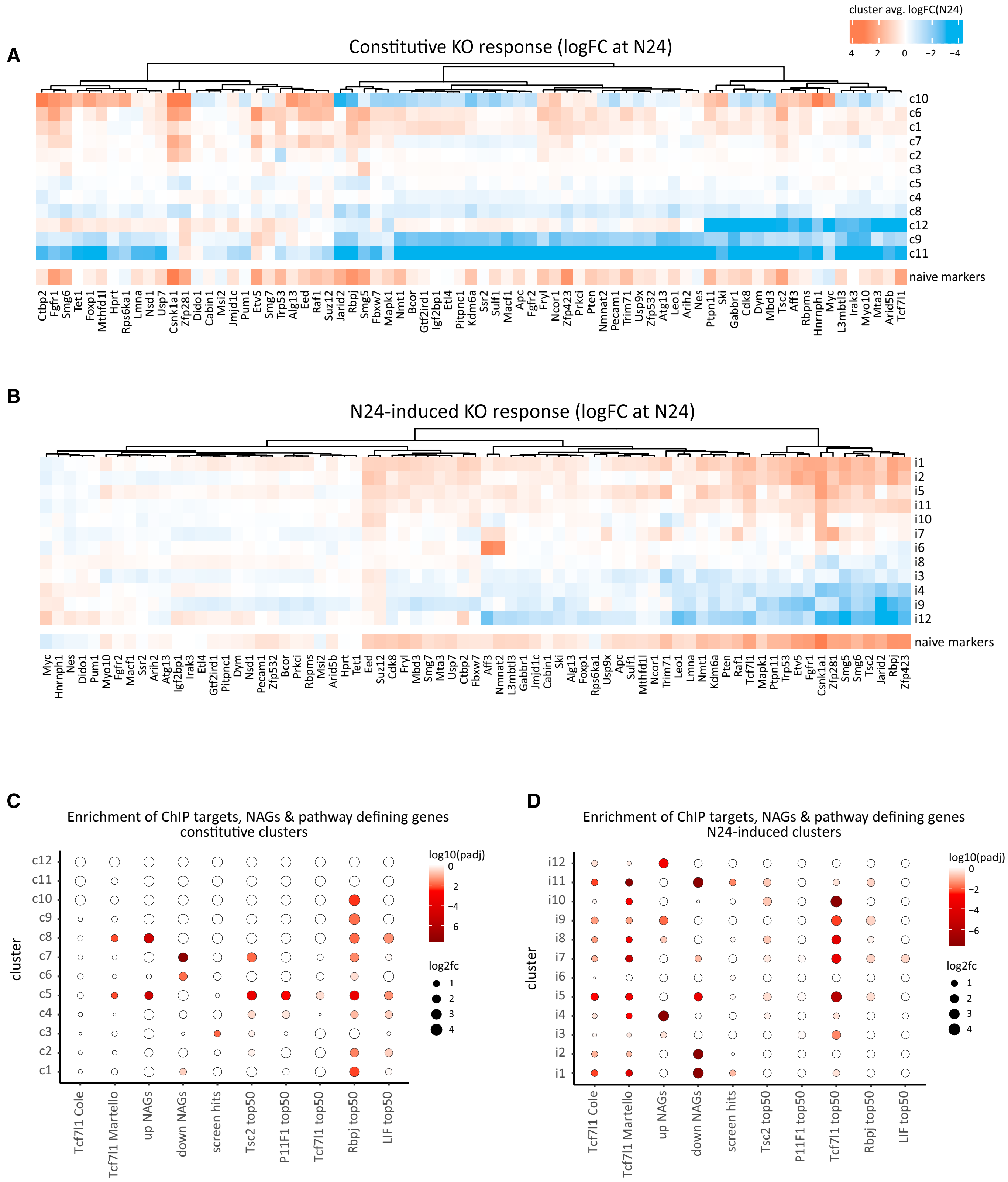

Figure EV4.

© 2021 The Authors

Figure EV5. Wiring between regulatory pathways and gene expression clusters.

A Schematic overview of the workflow to model cluster-pathway connections. White rectangles indicate input data from previous analysis steps, white ellipses indicate results from this analysis and blue rounded rectangles represent different processing steps and calculations to obtain indicated results. Schematic pathway to cluster relations over the time course is shown on the right. If the change in pathway activity is causal for a change in cluster expression, behaviour as shown in the top scheme is expected. The two schemes at the bottom exclude causality as the cluster changes precede changes in the pathway.

B Heatmap showing multiple regression models of pathway activities that predict cluster expression. Only significant (adjusted $P \leq 0.01$) interactions are shown. Colour intensity represents the strength of the interaction. Red tiles indicate that higher activity of a certain pathway is linked to higher expression of the cluster, while blue tiles indicate the inverse correlation.

C Observed cluster expression change (red) and cluster expression change predicted by regression models (blue) throughout the 32 h WT differentiation time course for all induced clusters. Predicted cluster expression is calculated using pathway activities throughout the WT time course and significant (adj. $P \leq 0.01$) interactions from the multiple regression models from (B). Graphs show log2FC of cluster expression relative to the mean cluster expression across the time course. Cluster–pathway connections that were not validated through this test (predicted expression did not follow the same trend AND/OR did not precede observed expression) were rejected, as indicated.

D Similar to Fig EV5C for constitutive clusters.

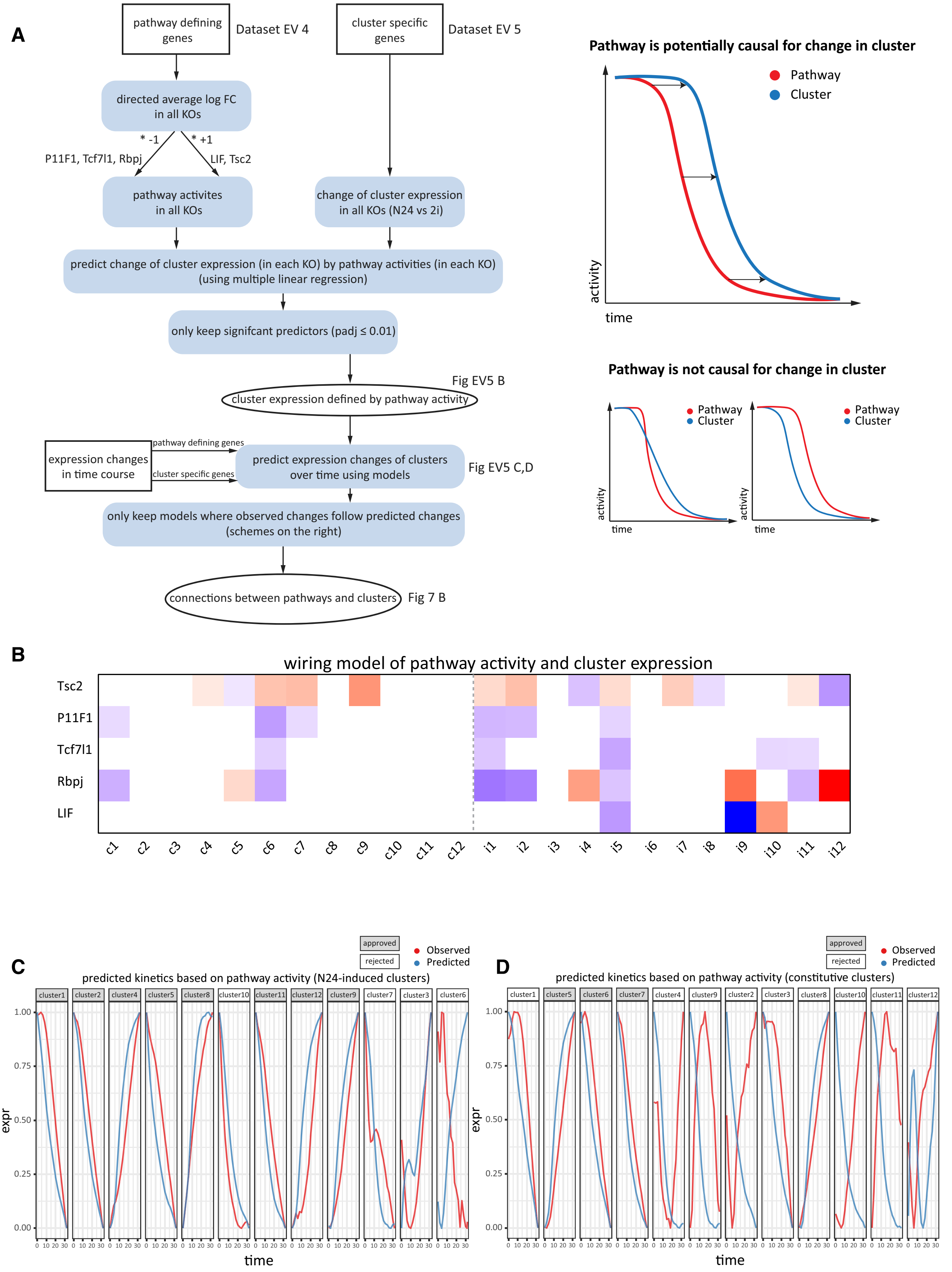

Figure EV5.

© 2021 The Authors
